# The Interplay of *cis*-Regulatory Elements Rules Circadian Rhythms in Mouse Liver

**DOI:** 10.1371/journal.pone.0046835

**Published:** 2012-11-05

**Authors:** Anja Korenčič, Grigory Bordyugov, Rok Košir, Damjana Rozman, Marko Goličnik, Hanspeter Herzel

**Affiliations:** 1 Institute of Biochemistry, Faculty of Medicine, University of Ljubljana, Ljubljana, Slovenia; 2 Institute for Theoretical Biology, Humboldt University, Berlin, Germany; 3 Center for Functional Genomics and Bio-Chips, Faculty of Medicine, University of Ljubljana, Ljubljana, Slovenia; Montana State University, United States of America

## Abstract

The mammalian circadian clock is driven by cell-autonomous transcriptional feedback loops that involve E-boxes, D-boxes, and ROR-elements. In peripheral organs, circadian rhythms are additionally affected by systemic factors. We show that intrinsic combinatorial gene regulation governs the liver clock. With a temporal resolution of 2 h, we measured the expression of 21 clock genes in mouse liver under constant darkness and equinoctial light-dark cycles. Based on these data and known transcription factor binding sites, we develop a six-variable gene regulatory network. The transcriptional feedback loops are represented by equations with time-delayed variables, which substantially simplifies modelling of intermediate protein dynamics. Our model accurately reproduces measured phases, amplitudes, and waveforms of clock genes. Analysis of the network reveals properties of the clock: overcritical delays generate oscillations; synergy of inhibition and activation enhances amplitudes; and combinatorial modulation of transcription controls the phases. The agreement of measurements and simulations suggests that the intrinsic gene regulatory network primarily determines the circadian clock in liver, whereas systemic cues such as light-dark cycles serve to fine-tune the rhythms.

## Introduction

Organisms have evolved biological clocks to adapt better to the 24 h period of the solar day. An endogenous circadian timing system controls daily rhythms in physiology, metabolism, and behaviour [1]. The mammalian circadian clock is a hierarchically organised system, coordinated by the bilateral suprachiasmatic nucleus (SCN) in the hypothalamus. The SCN neurons are entrained by light via the retinohypothalamic tract. Subsequently, the SCN orchestrates the rhythmicity of peripheral organs through hormonal signals, sympathetic enervation, and indirect cues, such as body temperature, feeding time and activity rhythms [2], [3].

At the molecular level, each cell has its own clock mechanism that is based on transcriptional feedback loops [4]. A set of about 20 core clock genes constitutes an intracellular gene regulatory network with multiple negative and positive feedback loops [5]. The rhythmic expression of genes is governed by central clock-controlled elements (CCEs), classified into morning, daytime, and night-time elements [6]. Extensive analyses of transcriptional regulation using an *in vitro* cell culture system revealed that primarily E-boxes, D-boxes, and ROR elements (RREs) control the rhythmic expression of genes [7]. The heterodimer BMAL1:CLOCK activates transcription of many clock genes through E-boxes [8]. Positive regulators DBP, TEF, HLF, and the negative regulator E4BP4 bind to D-boxes [9], [10]. In addition, clock genes are controlled by nuclear receptors ROR and REV-ERB through RREs [11].

With the aid of these clock-controlled elements, transcriptional feedback loops are formed in most mammalian cells [12]. E-box regulators such as *Period* (*Per1,2,3*) and *Cryptochrome* (*Cry1,2*) inhibit their own expression after some delay. In another negative feedback loop, the E-box driven gene *Rev-erb*a inhibits transcription of *Bmal1* via RREs. Both loops together constitute a robust intracellular oscillator [13].

In peripheral organs such as the liver, circadian rhythms are additionally affected by systemic cues: hormones, body temperature, metabolism, etc. The relative contributions of intracellular rhythms and systemic cues are not known [14]. Using a conditionally active liver clock [15], found 31 system-driven genes including *Per2*. In this paper, we test the hypothesis that the intracellular feedback loops dominate the circadian clock in liver.

We measured the time-resolved and normalised expression of 21 clock genes in mouse liver during light-dark (LD) cycles and constant darkness (DD) after entrainment of animals to alternate 12 h∶12 h light-dark cycles. Using these time-course data, we designed a minimal gene regulatory network that consists of six delay-differential equations (DDEs).

In order to reproduce the measured gene expression profiles using known *cis*-regulatory elements, we introduce a novel modelling concept in mammalian chronobiology: transcriptional regulation is controlled by time-delayed components. This approach allows to merge poorly characterised intermediate steps (post-translational modification, complex formation, nuclear localisation) into explicit delays of several hours. Delay-differential equations have been used in modelling of rhythmic [16] and even circadian processes [17], [18], but not explicitly for a model of mammalian circadian clock. In comparison to earlier models of mammalian circadian clocks without delay-differential equations [13], [19], [20], the number of model variables and parameters can be reduced drastically by such simplification.

The kinetics of transcriptional regulation is based directly on experimentally verified transcription-factor binding sites. The remaining unknown kinetic parameters are optimised to reproduce all of the experimentally measured phases and amplitudes. The high level of agreement between the experimental data and the simulations implies that a gene regulatory network driven by the E-boxes, D-boxes, and RREs is sufficient to reproduce complex expression patterns in liver.

We explore the interplay of *cis*-regulatory elements step by step. We start with a single self-inhibitory gene to provide a rigorous mathematical explanation of how time delays control onset and period of oscillations. A model with two interacting genes illustrates the interplay of activation and inhibition. Our final six-variable data-driven model, firmly rooted in experimentally known facts, allows us to study the necessary conditions for sustained oscillations, to understand the control of the phases via combinatorial regulation, and to find possible explanations for differences in gene expression in DD and LD regimes.

## Results

### Circadian expression profiles exhibit variable phases

Published expression profiles in mouse peripheral tissues depend on the measurement platform and the normalisation procedure [2], [3], [21], [22]. Our model is based on our own quantitative time-resolved RT-PCR data in mouse liver in constant darkness and under light-dark cycles. To obtain reliable amplitudes, we applied optimised normalisation through three reference genes (see [23] for details). The comparison of these novel high-resolution dark-dark and light-dark time-series allows to explore the role of systemic regulations of the liver clock.

The circadian gene expression profiles were obtained in C57BL/6J mice. The animals fed *ad libitum* were entrained for three weeks to 12 h∶12 h light-dark (LD) cycles, and then their gene expression was measured. For the DD experiments, LD-entrained mice were put into constant darkness 36 h prior to the measurements. The data were normalised using the reference genes *Hmbs*, *Eif2A*, and *Ppib* as described elsewhere [23]. Every 2 h, the expression of 21 genes was measured using at least 4 biological replicates.

**Figure 1:**
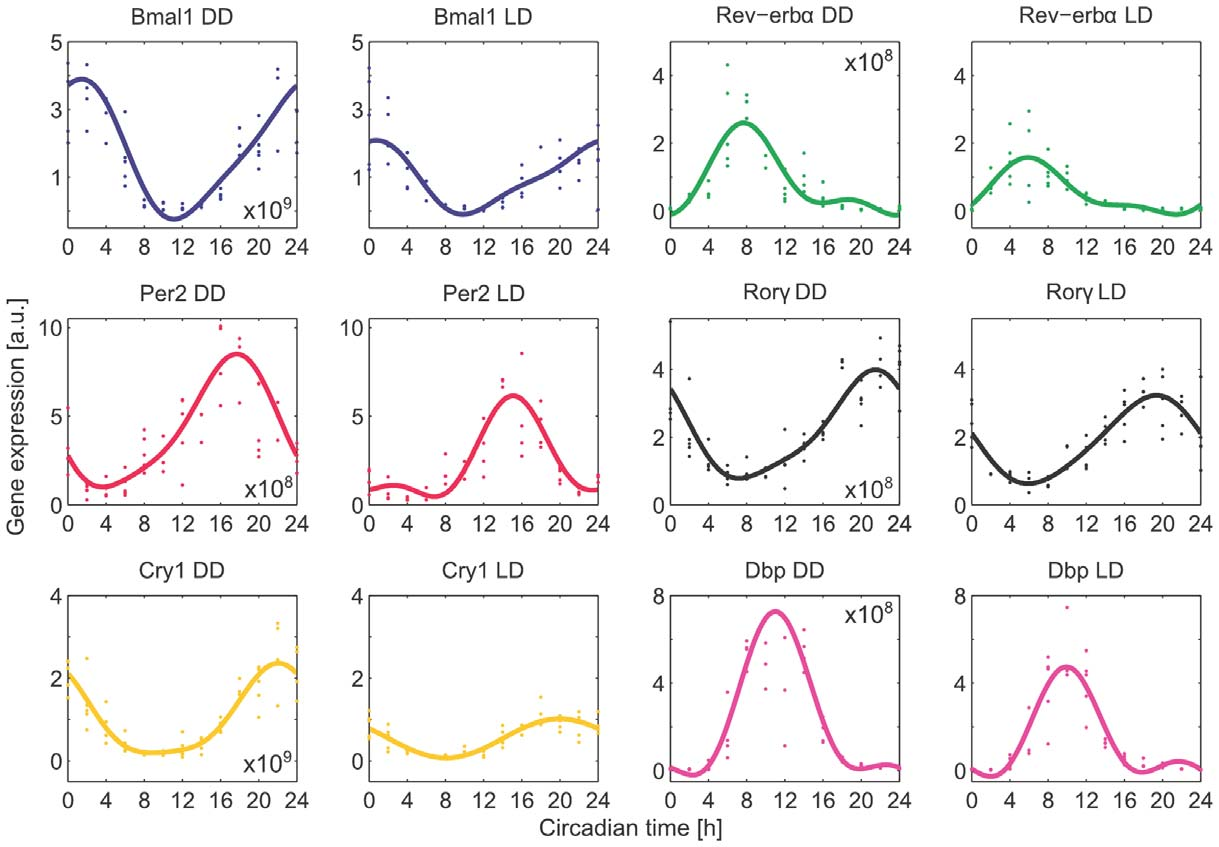
Gene expression of six core clock genes in mouse liver in constant darkness (DD) and 12 h∶12 h light-dark cycles (LD). Data were normalised by three reference genes and fitted by a function with 24 h and 12 h trigonometric terms (Equation (S1) in Supplementary Information S1 - Fitting of trigonometric functions to gene expression data).

The resulting time courses were fitted by sine and cosine functions. Figure 1 shows raw and fitted data. To fit variable waveforms (see Supplementary Information S1 - Fitting of trigonometric functions to gene expression data), we used sine and cosine functions with the main period of 24 h and two additional harmonics with 12 and 8 h. Based on the experimental data, we chose 6 out of 21 genes for our model of the core clock mechanism. Time-courses of the other 15 genes in DD and LD regimes are presented in the Supplementary Information S1 (Fitting of trigonometric functions to gene expression data). Peak phases, amplitudes, and waveforms of the 21 genes are quite variable, and there are clear differences between LD and DD regime measurements (Supplementary Information S1 - sections Parameters describing the oscillatory gene expression and Differences in DD and LD regimes). Our modelling approach aims to reproduce and explain the observed variability of phases and amplitudes. We start with just one self-inhibitory gene represented by a single delay-differential equation.

### A single negative feedback loop can lead to self-sustained oscillations

**Figure 2:**
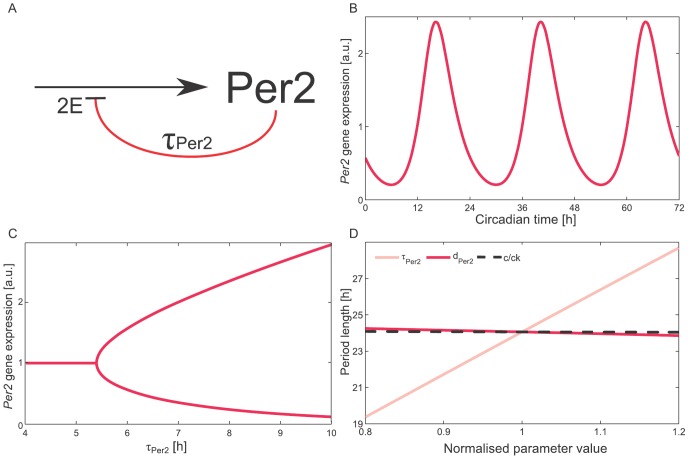
One-variable model - *Per2* self-inhibition. (A) Scheme of the one-variable model of self-inhibition of the clock gene *Per2* with explicit delay 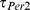 and two E-boxes (2E). (B) Observed delays and non-linearities provided by these two E-boxes (as described by Equation (1)) lead to 24 h oscillations. (C) Bifurcation analysis reveals oscillation onset at 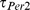 about 5.3 h. For larger explicit delays 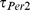, we plot maxima and minima of the oscillation. (D) Control of the period length for different parameters shows that the explicit delay has the strongest effect on the period. Parameter values for simulations: 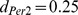 h-1; 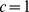; 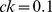; 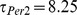 h. Gene expression in panels B and C is represented as normalised values divided by the mean of *Per2* expression.

To illustrate how delayed negative feedback loops can generate self-sustained rhythms, we model the self-inhibition of the clock gene *Per2*. The *Per2* regulatory region contains two E-box-like elements [24]. The protein product of *Per2* inhibits its own transcription through interactions with the BMAL1:CLOCK complex [4], whose abundance can be assumed to be constant [25]. We refer to Figure 2A for a graphical representation of our model of the delayed self-inhibition of *Per2*. Mathematically, this system can be modelled by a single delay-differential equation (DDE):
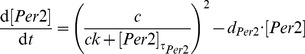
(1)


The exponent represents the number of CCEs in the *Per2* regulatory region [26]. Here, 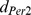 is the degradation rate of the *Per2* mRNA (taken from [27]), and 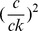 represents the basal *Per2* production rate. The subscript 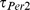 indicates that the value of *Per2* is taken at the time point 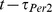, thus accounting for the delay including translation, post-translational modifications, complex formation, and nuclear translocation. According to published experimental data, this delay is about 8 h [28]. The other parameters in Eq. (1) can be fitted to provide 24 h oscillations of *Per2* of suitable amplitude.

Oscillatory behaviour can be achieved through overcritical delays [16] and sufficiently strong non-linearities [29] (see also Supplementary Information S2). Long half-lives can additionally retard inhibition and thus contribute to the total delay between production and inhibition [30], [31], (see Supplementary Information S3 - Long half-lives shift expression peaks). Mathematical analysis of the system shows that the period of the emerging oscillations is two to four times the explicit delay (Supplementary Information S2). In our model, the explicit delay of 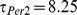 h resulted in oscillations with a period of 24 h (Figure 2B). The relatively long delays predicted by our mathematical analysis point to the essential role of post-translational modifications, complex formations, and controlled nuclear translocation in the circadian system.

Figure 2C illustrates the emergence of oscillations under variation of the parameter 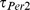. For the given set of parameters, the critical value of the explicit delay 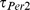 is around 5.3 h. At this point, a Hopf bifurcation occurs – a transition in which the stationary point of our system loses stability, and a limit cycle with small amplitude emerges. An increase of delay parameter leads to an increase of the amplitude of *Per2* oscillations.

Figure 2D also shows that an increase of 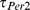 results in an increase of the oscillation period. The period decreases with increasing degradation rate of *Per2*, i.e. longer half-lives result in larger periods. In comparison to the effect of explicit delay and degradation rate, the effect of the production term on the period length is quite small. Close to the Hopf bifurcation, an expression for the dependence of the oscillation period on the parameters of the equation can be derived rigorously (see Supplementary Information S2). In particular, the oscillation period is proportional to the time delay 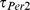.

Summarising, this model shows that even one-variable DDE models can lead to 24 h oscillations, provided that the system is non-linear and has sufficiently long delays. However, more complex models are needed to explore the other feedback loops and phase differences between the different clock components.

### The nuclear receptor loop as a potential oscillator

**Figure 3:**
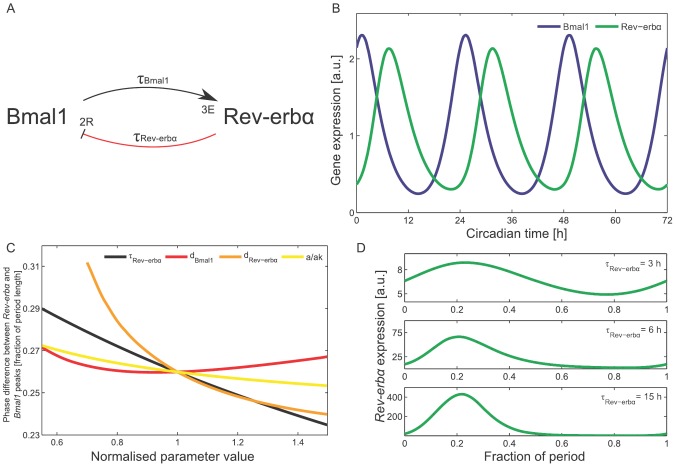
Two-variable model - nuclear receptor loop. (A) Scheme of the two-variable model of the *Bmal1* - *Rev-erb*a loop showing the number of relevant CCEs of each gene (2R - two RREs; 3E - three E-boxes). (B) Simulations show that 24 h oscillations with a correct phase difference can be generated by using experimentally observed explicit delays and non-linearities arising from the number of CCEs. (C) The phase difference between the two genes is effected by model parameters with different strength. (D) The waveform is controlled by the explicit delays; greater delays lead to sharper peaks. Parameter values for simulations: 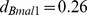 h-1; 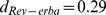 h-1; 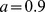; 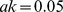; 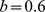; 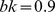; 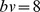; 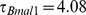 h; 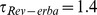 h. Gene expression in panels B and C is represented as normalised values divided by the mean of the expression of the corresponding gene.

In addition to the negative feedback loop with *Per* and *Cry* genes, another loop with the nuclear receptor *Rev-erb*a has been discovered [11]. In [11] and [32], it was described that BMAL1 activates *Rev-erb*a via E-boxes, and how this nuclear receptor inhibits *Bmal1* gene expression (Figure 3A). *Bmal1* has two ROR-elements (RREs), and *Rev-erb*a is activated through three E-boxes [3], [11]. This system can be described with the following DDEs:
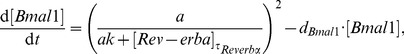
(2)

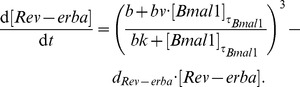
(3)


Here, 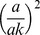 and 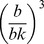 represent the basal transcription rates of *Bmal1* and *Rev-erb*a, and 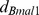 and 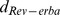 are the corresponding degradation rates. The parameter 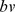 scales the *Bmal1* activation via E-boxes. The first term on the right-hand side of Equation (3) describes the transcriptional activation of *Rev-erb*a via *Bmal1* after a delay 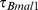. In Equation (2), *Rev-erb*a inhibits *Bmal1* transcription after a delay 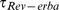. Again, the exponents in the production terms represent the number of relevant regulatory elements in the regulatory regions of the genes.

Data on mRNA and protein accumulation in liver nuclei from [11] suggest an explicit delay of *Rev-erb*a of 2 h and a 4 h delay of *Bmal1*. The degradation rates were chosen in the range of published values [27], [33]. Other parameters have been fitted to provide 24 h oscillations with a correct phase difference between the two genes according to our experimental data (Figure 1).

Although the interaction of *Bmal1* and *Rev-erba* is often considered as an auxiliary stabilising feedback loop, the observed delays [11] and the non-linear inhibition can generate self-sustained oscillations (Figure 3B, see also [13], [20]). The resulting phase difference between *Bmal1* and *Rev-erb*a is important to understand the interplay between those regulatory factors. Figure 3C explores how different parameters govern the phase difference. We found that for the chosen parameter set, the delay and the degradation rate of *Rev-erb*a have the strongest effects on the phase difference between the genes. The basal transcription rates of the genes was found to have only minor effects on the phase difference between the two genes.

In Figure 3D we show that larger explicit delays lead to sharper peaks. This finding is important for fitting the model to the data. However, we can control the peak width only to a certain extent, as the changes in explicit delays lead to changes in period length.

In summary, simulations of our two-variable model reveals that the experimentally observed delays and regulatory elements are sufficient to generate self-sustained oscillations with the negative *Rev-erb*a - *Bmal1* feedback loop.

### A six-variable model reproduces period, phases, and amplitudes of the core clock

Above, the role of explicit delays, degradation rates, and production terms was investigated in the sub-models of the *Per2* and *Bmal1*-*Rev-erb*a loops using reasonable kinetic parameters and known CCE regulation. These models need to be extended to provide an in-depth understanding of the interplay of the regulatory loops of the core circadian clock. A simple three-variable model with the core clock genes *Per2*, *Bmal1*, and *Rev-erb*a failed to reproduce the experimentally observed phase of *Per2*. In order to solve this problem, we added *Dbp* as an additional regulator acting via D-boxes. Moreover, we included *Cry1* and *Rorg* as important clock genes in the liver with large amplitudes and characteristic late phases (see Figure 1).

**Figure 4:**
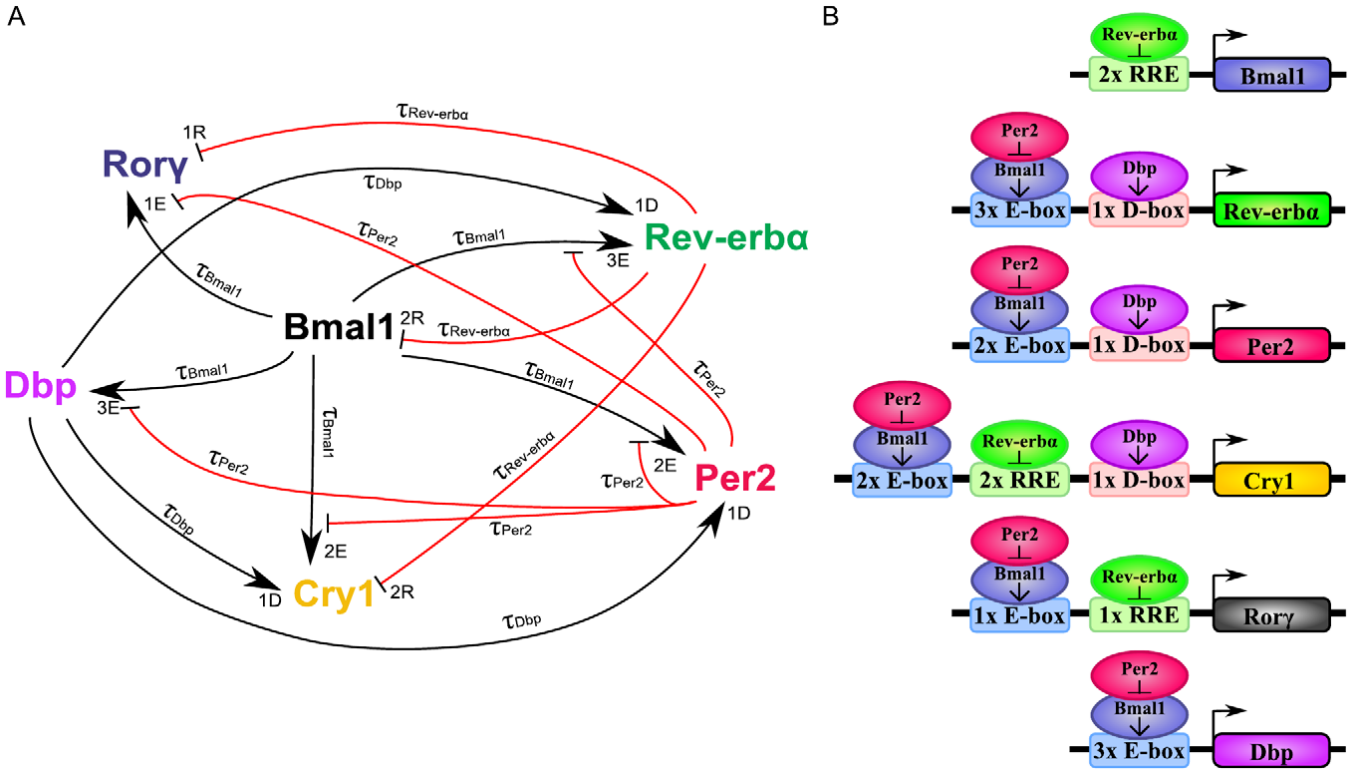
Six-variable model of core clock. (A) Model network containing six clock genes. The numbers of the CCEs are shown next to the arrowheads for each gene (E, E-box; D, D-box; R, RRE) and the explicit delays are noted at the arrows. (B) Regulatory regions containing the CCEs are shown for the six genes included in the model. Experimental evidence for the CCEs is given in Supplementary Information S4 - Clock-controlled elements.

The final six-variable model contains *Bmal1*, *Rev-erb*a, and *Per2*, together with *Cry1*, *Rorg*, and *Dbp* (see Figure 4). The expression regulation of each gene is modelled solely at the transcriptional level. Explicit delays represent the time span that is needed for protein synthesis, modifications, translocation, and complex formation. Many of these intermediate reactions that involve numerous post-translational modifications are not well characterised. Thus, the concentrations of the active proteins are approximated through the delayed concentrations of mRNAs. The model is based on minimal assumptions regarding transcriptional regulation: the production terms are derived from the CCEs (Figure 4B) of each gene in the same manner as in the simpler one-variable and two-variable models. Linear degradation is assumed for all mRNAs.

The resulting clock model is described by a system of six DDEs:
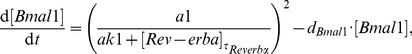
(4)

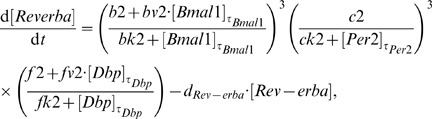
(5)

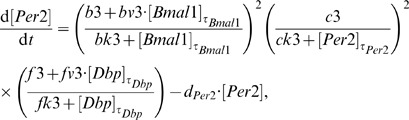
(6)

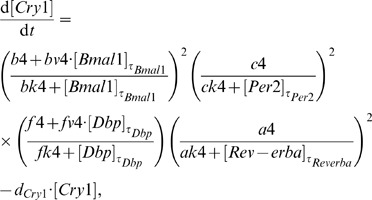
(7)

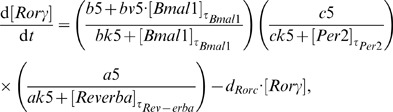
(8)

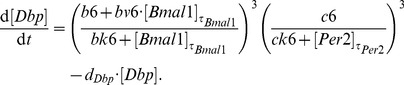
(9)


For each gene, its dynamics is controlled by a production term, accounting for the transcriptional regulation, and a degradation term with the corresponding degradation rate. The production term of each gene is a product of brackets. Each bracket, which we term “modulation factor” in the following, accounts for the contribution from the corresponding CCE. Every modulation factor is constructed in the same way for all genes, although the final parameter values can differ due to different regulatory strength (see Supplementary Information S4 - Parameter estimation). Regulation by different CCEs is considered to be independent, leading directly to the multiplication of modulation factors [26].

If the corresponding concentrations of the regulatory factors vanish, we obtain the basal transcription rates (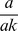 for RRE, 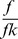 for D-boxes, and 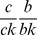 for E-boxes). The powers in the equations represent the numbers of experimentally verified binding sites as listed in the Supplementary Information S4 (section Clock-controlled elements).

The choice of degradation rates 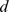 and explicit delays 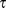 is based on published experimental data (see Supplementary Information S4 - Parameter estimation). The remaining parameter values have been optimised to reproduce phases, amplitudes, and waveforms of our measured expression profiles (see Supplementary Information S4 - Parameter estimation).

**Figure 5:**
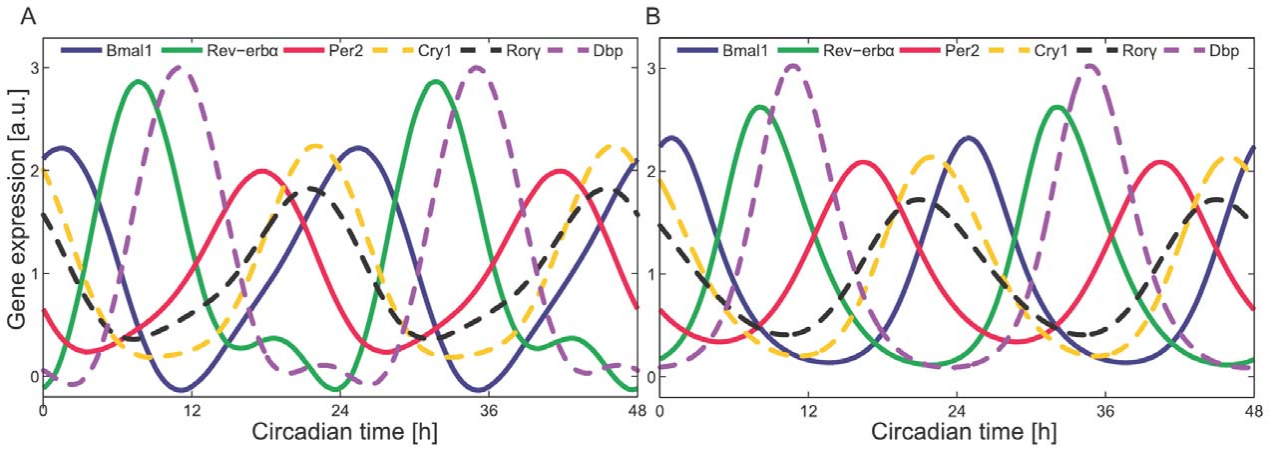
Comparison of experimental data and the six-variable model. (A) Fitted experimental gene expression data of the six core clock genes in mouse liver for the DD regime. (B) Our model (Equations (4) to (9)) reproduces the experimental data for the period length, phases, amplitudes and waveforms of these core clock genes. In both panels, gene expression is normalised by dividing by the mean expression of the respective gene.

We emphasise that our model is a straightforward representation of a minimal gene regulatory network. This network includes just three clock-controlled elements (E-box, D-box, and RRE) and no systemic regulation. Despite this simplicity, the model reproduces the experimentally measured phases, amplitudes, and waveforms (see Figure 5).

In Figure 5 we compare the experimental data (left panel) with model simulations (right panel). In both cases, *Dbp* has the largest and *Rorg* the smallest amplitude. The simulations reproduce the phases of the six genes, spanning the whole period of 24 h: *Bmal1* has the earliest peak, at about CT 1, followed by *Rev-erb*a (maximum at about CT 8) and *Dbp* (at CT 11). We achieved that *Per2* has its maximum at CT 17, and reproduced quite late phases of *Rorg* (CT 21) and *Cry1*, which is consistent with previous experimental studies [3], [8], [21], [22]. The simulated peak widths also agree with the measured expression profiles: narrow peaks for *Rev-erb*a and *Dbp* and broad peaks for *Rorg*.

Our model is a direct translation of experimental observations to DDEs composed of production and degradation terms. The production terms consist of modulation factors which represent individual clock-controlled elements. In the following sections, we discuss how the interplay of modulation factors explains the combinatorial regulation of clock genes.

### Delayed *Rev-erb*a inhibition determines *Bmal1* phase

**Figure 6:**
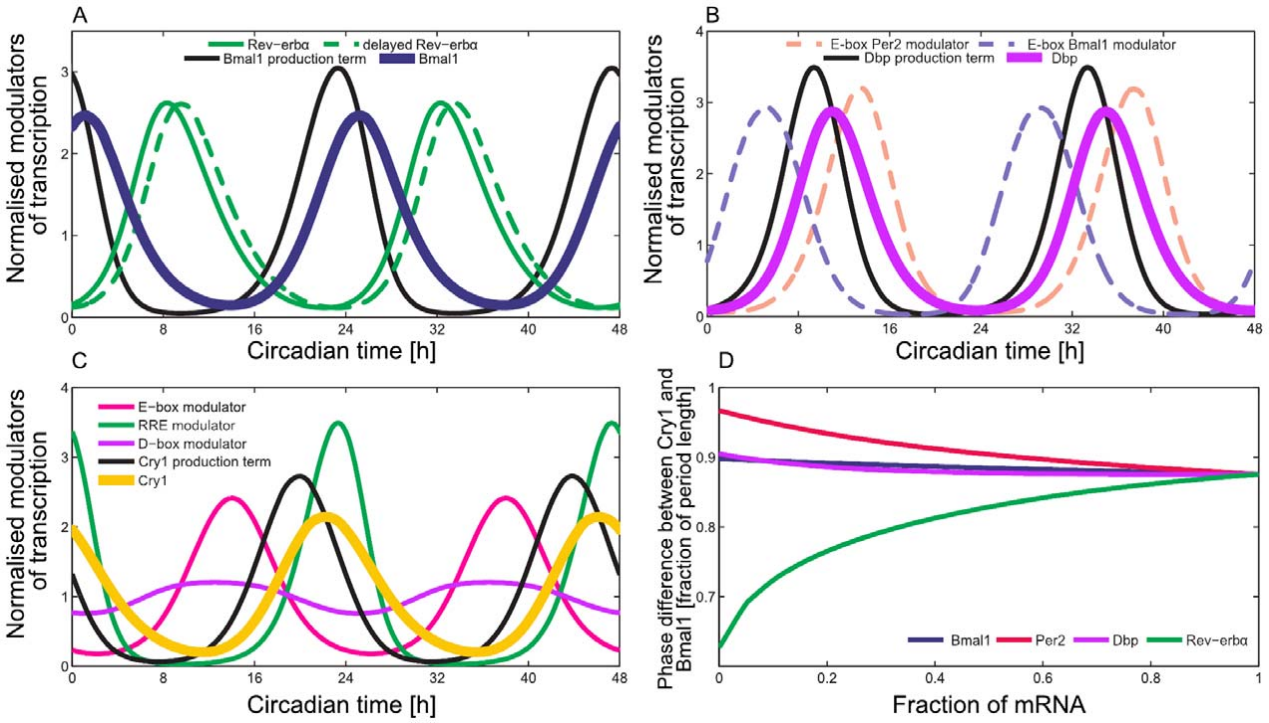
Regulation of phase variability. Thick lines in panels A, B, C refer to the corresponding mRNA and black lines mark the production terms; coloured lines in B and C represent “modulation factors”, see text. Long half-lives lead to later peaks of mRNAs (thick lines) compared to production terms (black lines). (A) Delayed *Rev-erb*a inhibits *Bmal1* expression. (B) *Per2* and *Bmal1* modulators determine *Dbp* production. (C) RRE modulator (green) and E-box modulator (red) govern *Cry1* production. (D) Reducing the amounts of regulators *Bmal1*, *Per2*, *Dbp*, and *Rev-erb*a mimics RNAi experiments and knock-outs. The resulting phase shifts agree with experimental data (see text).

The expression of *Bmal1* is a simple example for phase control, since it is regulated by a single modulation factor, see Equation (4). Figure 4B shows that *Bmal1* expression is modulated by nuclear receptors *Ror* and *Rev-erb* through two RREs. The green line in Figure 6A indicates the mRNA oscillation of *Rev-erb*a that peaks at CT 8. The dashed green line represents the delayed mRNA acting as an inhibitor of *Bmal1* transcription. According to Equation (4), the *Bmal1* production term (Figure 6A, black line) oscillates out of phase to the inhibitor *Rev-erb*a. Eventually, the mRNA peak of *Bmal1* (thick blue line in Figure 6A) is further delayed by about two hours due to its half-life of 2 h (see [34] and Supplementary Information S3 - Long half-lives shift expression peaks).

Summarising, we established three main factors that contribute to the 17 h phase difference between *Rev-erb*a and its target gene *Bmal1*. The explicit delay of *Rev-erb*a contributes about an hour. The production term is further delayed by 14 h, since its peak coincides with the minimum of the inhibitor *Rev-erb*a. Finally, the half-life of *Bmal1* adds about two more hours to the total phase difference of 17 h, assuming that the delay is approximately proportional to the half-life (see Supplementary Information S3 - Long half-lives shift expression peaks). This example illustrates how our quantitative model of non-linear regulation explains the observed phases.

### Positive and negative E-box modulators dictate *Dbp* phase

The phase control of *Dbp* via E-boxes is more complex, since its expression is regulated both positively by *Bmal1* and negatively by *Per2*. The phase of the *Bmal1* E-box modulator (blue dashed line in Figure 6B) lags behind the *Bmal1* phase, which is an effect of the explicit delay 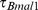 of 4 h. The phase of the *Per2* E-box modulator (dashed orange line in Figure 6B) lags behind the anti-phase of the *Per2* expression by 8.4 h (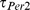). The phase of the *Dbp* production term (Figure 6B, black line) is between the phases of the *Bmal1* and *Per2* E-box modulators, since the amplitudes of both modulators are comparable. Using trigonometric approximation, which is applicable here due to the simplicity of waveforms, we show in Supplementary Information S3 (Multiplying transcriptional modulator factors) that the combination of the modulation factors with similar amplitudes leads to an intermediate phase of the total production term. In addition, the expression of *Dbp* itself is delayed according to its half-life (thick line in Figure 6B).

This example introduces the interplay of two antagonistic regulators in the determination of the output phase, and illustrates the concept of synergy between activators and inhibitors – a common motif of the circadian clock. Several activator-inhibitor pairs have been described in greater detail: *Ror*-*Rev-erb* [11], [35], *Dbp* -*E4bp4* [10], and *Bmal1* -*Dec1* [36]. mRNAs and proteins of activators and inhibitors are often expressed at opposite phases – the maximum of activation coincides with the minimum of the inhibition. Thus larger amplitudes of target genes are expected [7], [37]. As shown in Supplementary Information S3 (Synergy of antiphase activators and inhibitors), antiphase activators and inhibitors can be modelled using a single regulator with enlarged amplitude. Therefore, the *Rorg* and *E4bp4* effects are implicitly included in our *Rev-erb*a and *Dbp* terms, respectively.

For E-boxes, such a simplification does not apply. E-box regulation is governed by a combinatorial effect of at least 11 genes (*Bmal1*, *Bmal2*, *Clock*, *Npas2*, *Cry1*, *Cry2*, *Per1*, *Per2*, *Per3*, *Dec1*, *Dec2*) together with numerous post-translational modifications as elegantly shown by [5]. In our model, two separate E-box regulators (*Bmal1* and *Per2*) are used to represent at least some of the complexity of E-box regulation.

### Combinatorial control of delayed *Cry1* expression

As illustrated in Figure 4B, the late *Cry1* expression is regulated by multiple factors. In earlier studies, the phases of the clock genes were modelled through the superposition of trigonometric functions [7], [38], or by simply predicting them from the CCEs [3]. We exploit our model to understand the regulation of phases by multiple regulators in detail. The phase of *Cry1* expression is determined by all three circadian CCEs through the action of four genes (*Bmal1*, *Per2*, *Rev-erb*a, *Dbp*). The amplitudes of the three modulators are quite different. Simple trigonometric considerations (see Supplementary Information S3 - Multiplying transcriptional modulator factors) support the intuition that the largest amplitudes dictate the resulting phase. Consequently, the *Cry1* production term (black line in Figure 6C) is in between the E-box modulator (red line in Figure 6C) and the RRE modulator (green line in Figure 6C). Figure 6C shows that the net expression of *Cry1* gene is even more delayed due to its relatively long half-life.

We performed simulations that mimic RNA interference experiments and monitored their effects on the *Cry1* phase. The results in Figure 6D show the effects of knock-downs of regulatory genes. The left side of Figure 6D (where the fraction of mRNAs is equal to zero) represents the effects of the knock-out of the corresponding modulators. As shown experimentally, removal of *Cry1* RREs causes a phase advance of *Cry1* ; the deletion of both of the RREs from the intronic region of *Cry1* resulted in a 3.7 h phase advance of *Cry1* [38]. Our simulations show a phase advance of about 5 h. In [38], the clock gene expression profiles were also measured in *Dbp* ^−/−^:*Tef* ^−/−^:*Hlf* ^−/−^ mice and a delay of *Cry1* was found. This is also in agreement with our simulations, where the knock-down of *Dbp* causes a phase delay of about 1 h.

### *Per2* dynamics strongly controls the system

E-box regulation constitutes a hub of the circadian gene regulatory network [7]. Perturbation analysis of our model allows to find the most sensitive parameters (Supplementary Information S5). The period length is strongly affected by parameters related to *Per2* dynamics, which is consistent with previous studies [39]. The strongest effects are induced by changing the explicit delay 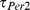 similarly to the one-variable and two-variable models.

Explicit delays have strong influences on period length, phases control, and amplitudes of all genes. Analysing the two-variable model, we concluded that for the chosen parameter set, 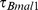 is most important for generation of oscillations, whereas 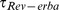 is responsible for the phase difference between the two genes. In our six-variable model, 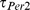 is the most important delay for the control of phase and period length, and 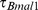 and 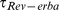 fine-tune these properties.

The delay 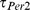 is strongly influenced by phosphorylation and nuclear translocation [40], [41]. In particular, casein kinases are important for the maintenance of the 24 h period [42]. This observations agree with our model, showing sensitivity with respect to *Per2*. Control analysis of a previously published model [19], [39] reached an analogous conclusion.

We also checked how changes in the overall transcription rate influence the system (see Supplementary Information S5 and Spreadsheet S1). A 10% change in the overall transcription rate results in a 0.2% shorter period length. Although minor effects on the phases of the clock genes are observed, the order of the peaks remains unchanged. This is in agreement with the findings from a study of [43], who claimed that circadian gene expression is resilient to fluctuations in the overall transcription rates.

### Explaining phase and amplitude changes due to light-dark entrainment

**Figure 7:**
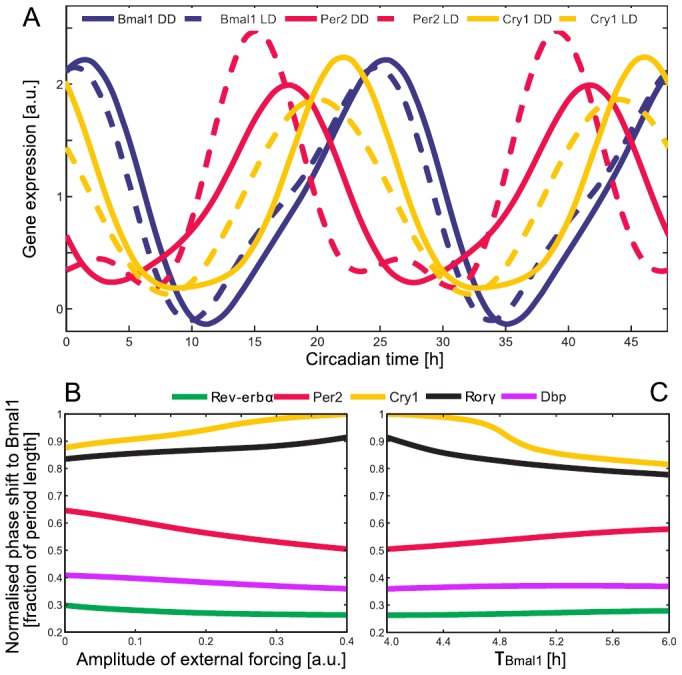
Experimental data and model in DD and LD regimes. (A) Normalised gene expression of *Bmal1*, *Per2*, and *Cry1* under DD and LD regimes. (B) Adding a 12 h∶12 h step function that modulates *Per2* transcription increases the *Per2* amplitude (not shown) and causes phase advance of all genes except *Rorg* and *Cry1*. (C) Starting from the endpoint in panel B, increasing the explicit delay of *Bmal1* 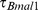 leads to correct *Rorg* and *Cry1* amplitudes and phases without substantially changing the dynamics of the other genes.

A careful investigation of the DD and the LD experimental datasets shows that there are significant differences (see Supplementary Information S1 - Differences in DD and LD regimes). Figure 7A compares the expression of *Bmal1*, *Cry1* and *Per2* in LD and DD regimes. As discussed above, E-boxes constitute a hub of circadian regulation [7], [44]. Since *Bmal1* expression is not drastically changed in DD and LD regimes, *Per2* appears to account for many of the changes in the system. Under DD conditions, we observe phase advances in all of the core clock genes included in our model (Supplementary Information S1 - Differences in DD and LD regimes).

We have exploited our control analysis (Supplementary Information S5) to identify the parameters that can cause the observed differences between liver gene expression in DD and LD regimes. The most promising candidate is the parameter 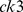 (connected to the strength of the self-inhibition of *Per2* transcription; see Equation (6)). This parameter affects all amplitudes and phases via an increase of *Per2* amplitude.

Alternatively, the increased amplitude and the earlier phase of the *Per2* oscillation in LD can be explained considering an external driver as suggested by [15]. Through the construction of a conditionally active liver clock, in [15], it was shown that *Per2* is driven by systemic cues. We modelled such systemic cue as a 12 h∶12 h step function that modulates *Per2* transcription and thus influences its amplitude. An increase in the strength of the external forcing increases the *Per2* amplitude and causes phase advances as seen in our experimental data (Figure 7B). Interestingly, changing parameter 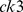 and the additional drive by the systemic cue lead to surprisingly similar effects. This indicates that an increase of *Per2* amplitude is a major effect of light-dark cycles.

However, the effects of these parameter changes on *Cry1* in our simulations do not align with our experimental data. We attribute this discrepancy to the complex regulation of *Cry1* (see Figure 4B). We used control analysis and found that 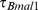, 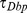, 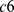, and 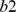 were most promising candidates for correcting the phase and the amplitude of *Cry1*.

Numerical analysis reveals that changes in 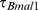 can explain the earlier phase of *Cry1* in the LD regime (Figure 7C). The value of 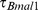, adapted to the LD situation, still belongs to the experimentally determined range (Supplementary Information S4 - Parameter estimation). The dynamics of the *Bmal1* gene is controlled by *Rev-erb*a (Equation (4)). It is known that *Rev-erb*a expression is regulated by metabolic processes [45] that differ between DD and LD regimes. Consequently, differential regulation of *Bmal1* could induce a longer delay 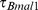 in the LD regime. Thus it seems plausible that different physiological conditions in LD lead to a longer delay.

## Discussion

The architecture of the core clock has been deciphered in the past decade [4], [46]. Delayed negative feedback loops generate oscillations in almost all mammalian cells. With the aid of reporter constructs, the transcriptional circuits underlying the circadian clock have been identified in cell cultures [7]. In this paper, we have shown that the proposed gene regulatory network reproduces gene expression patterns in the liver tissue under DD and LD conditions. The quantitative agreement of expression data and numerical simulations suggest that the intracellular feedback loops govern the liver clock.

### Design of a minimal gene regulatory model

A comprehensive model of the mammalian clock requires quantitative details of post-transcriptional modifications, complex formation, nuclear translocations, and epigenetic regulations [47]. Since many of these kinetic processes are poorly characterised, we decided to condense these events into explicit delays between gene transcription and regulatory function. The duration of the delays can be estimated from the protein measurements as outlined in the Supplementary Information S4 (section Parameter estimation).

Our model is simplified by making minimal assumptions for production and decay. We assume linear degradation kinetics and take half-lives from the literature [27], [33], [48]. Transcription terms are derived from basic thermodynamics principles [26] and the number of the *cis*-regulatory clock-controlled elements is based on experimental data. The remaining unknown parameters are tuned systematically to reproduce measured amplitudes and phases (see Supplementary Information S4 - Parameter estimation). For parameter estimation, we could exploit the amplitude information, since the underlying time-resolved RT-PCR data have been carefully normalised.

### Selected genes represent activator/inhibitor pairs

We demonstrate that a network of a relatively small subset of core clock genes can reproduce rhythmic gene expression. Even the simplest one- and two-variable DDE models result in oscillations. Our final model describes time-dependent expression of six genes: *Bmal1*, *Rev-erb*a, *Per2*, *Cry1*, *Rorg* and *Dbp*. These components of the core clock can also implicitly represent other genes.

As a representative of the E-box negative regulators, we included *Per2* in our model. Although *Per1,2* and *Cry1,2* contribute to the transcriptional inhibition of E-boxes as well, we settled upon *Per2* for the following reasons: *Per1* exhibits a smaller amplitude than *Per2* (Supplementary Information S1 - Fitting of trigonometric functions to gene expression data) and it is the changes in PER2 degradation and nuclear translocation that have profound effects on the period of oscillations [40], [47]. PER2 is also involved in the familial advanced sleep phase syndrome (FASPS, see [49]). Moreover, PER seems to be the rate-limiting component for PER:CRY complex formation [50], [51]. These experimental facts justify the choice of *Per2* as a representative inhibitor of E-boxes.

The long explicit delays of *Bmal1* and *Per2* reflect complex formations with CLOCK, NPAS2, and CRY, post-translational modifications, and nuclear translocation. Furthermore, anti-phasic inhibitors (DEC for BMAL1, E4BP4 for DBP) or anti-phasic activators (ROR for REV-ERB) are implicitly represented through the different parameters values of the corresponding production terms (see Supplementary Information S3 - Synergy of antiphase activators and inhibitors). Such a synergy of activator-inhibitor pairs appears to be a common design principle of the circadian regulators. In this manner, large amplitudes of circadian gene oscillations can be achieved as discussed earlier [7], [37].

### Interplay of *cis*-regulatory elements determines phases

The phases of clock-regulated genes span the entire 24 h range [2], [3], [21], [22]. Our computational model clarifies the control of the phase differences between transcription factors and their target genes. Several contributions add up to the total phase differences: the explicit delay, the effects of the non-linear production terms, and half-life-related delays. For instance, *Bmal1* has its expression peak at CT 1, whereas many E-box-regulated genes in liver have a peak at about CT 10 [8]. Long half-lives lead to the peak phases that are up to 6 h delayed with respect to their transcriptional up-regulation (see Supplementary Information S3 - Long half-lives shift expression peaks, discussed also in [34]).

In order to determine the phase of transcriptional up-regulation we studied the complex interplay of the modulation factors in detail. These modulation factors refer to specific regulators such as BMAL1, PER2, REV-ERBa, or DBP. The product of these periodic modulators can be approximated by a product of trigonometric functions representing individual modulators (Supplementary Information S3 - Multiplying transcriptional modulator factors). The E-box regulation, for example, has its peak phase between the phases of BMAL1 and PER2 modulators (see Figure 6B). The late phase of *Cry1* is dictated by the large amplitudes of the RRE and the E-box modulators (see Figure 6C). The D-box modulator has a smaller amplitude and, hence, only a minor effect on the *Cry1* phase. Simulated down-regulation of clock gene concentrations agrees with RNAi experiments [44] and knock-out data [38]; see Figure 6D.

Our concept of phase-specific transcriptional modulators can be applied to predict the phases of genes based solely on their clock-controlled elements. The CT 22 phase of the RRE modulator is in anti-phase to the delayed *Rev-erb*a gene (see Figures 6A and 6C). The D-box modulator has its peak about CT 12, which corresponds to the delayed *Dbp* gene. The E-box regulation is itself controlled by two modulators; the *Bmal1* modulator peaks like the delayed *Bmal1* gene at CT 5. The *Per2* modulator is in anti-phase with the delayed *Per2* gene and peaks at CT 15 (see Figure 6B). The distinct peaks of these modulators imply that the target genes of the E-box regulation can be induced in the range from CT 5 to CT 15. This is due to the fact that a product of modulators can exhibit peaks in between their individual phases (Supplementary Information S3 - Multiplying transcriptional modulator factors). Furthermore, the phase of target genes can be delayed by a few hours if their half-lives are long (Supplementary Information S3 - Long half-lives shift expression peaks).

### Prediction of phases of clock-controlled genes

Our framework of transcriptional modulators predicts specific phase ranges for target genes: the dominance of E-boxes may lead to peaks of target gene expression in the range of CT 6 to CT 13, the combination of E-box and D-box regulation should broaden the range to CT 6–18. If the RREs dictate the phase, CT 0–6 can be expected. A combination of the RREs with the D-boxes and the E-boxes may lead to phase shifts in both directions.

**Figure 8:**
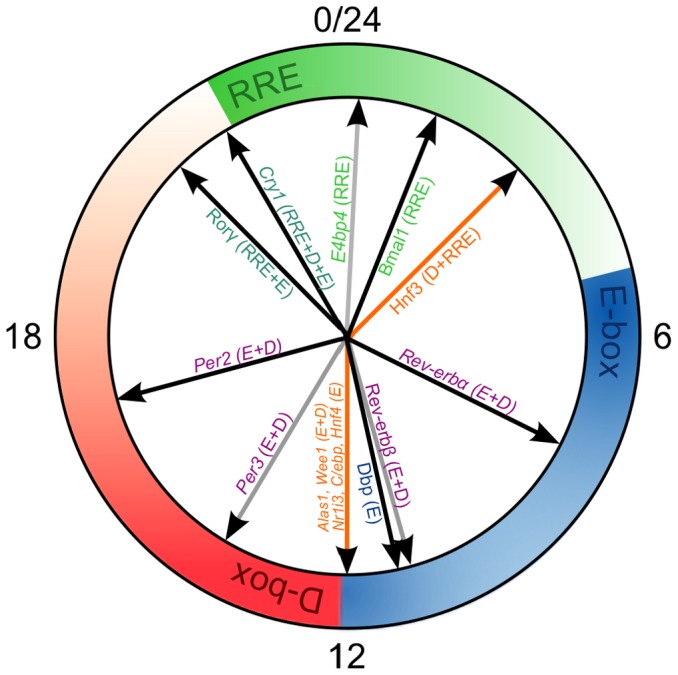
Phases of clock and clock output genes in DD. The outer circle represents the regions of the predicted expression phase of the clock genes that are regulated by the three CCEs included in our model. The phases of the genes from our model are depicted with black arrows, to serve as guidelines for other genes. Some clock genes that we measured in our experimental setup are also included with gray arrows. The phases of additional genes have been extracted from [22] and are shown with orange arrows.

We applied these principles to predict the phases of liver-specific clock genes in the DD regime by taking into account their transcription factor binding sites (using DECODE database, SABiosciences). First, we consider the genes that were measured in our experimental setup and are shown in the Supplementary Information S1 (Fitting of trigonometric functions to gene expression data). Figure 8 displays these genes with gray arrows. As predicted, *Per3* and *Rev-erb*b, which are targets of the E-box and D-box regulation, peak at about CT 12, whereas *E4bp4*, regulated by an RRE, peaks at CT 0.

The orange arrows in Figure 8 refer to additional genes with pronounced circadian rhythms according to the Circa database [22]. *Hnf3* is regulated by D-boxes and RREs, and it has a peak phase at CT 3. This indicates that, as in many other cases, the RRE modulator has a larger amplitude than the D-box modulator. For *Nr1i3*, *C/ebp*, and *Hnf4*, the E-box regulation leads to peaks about CT 12. Targets of E-boxes and D-boxes such as *Alas1* and *Wee1* also have expression peaks at CT 12. Again, the D-box modulation factors seem to induce no major phase shifts in these cases. These relatively weak effects of D-boxes on the clock genes are consistent with subtle alteration of phenotypes of the *Dbp* ^−/−^:*Tef* ^−/−^:*Hlf* ^−/−^ triple knock-out mice [52].

### Differences between constant darkness and light-dark cycles

The liver does not receive light input directly. Instead, the phase of the liver clock is adjusted by systemic cues such as hormones, body temperature, and feeding rhythms. Accordingly, we found significant differences between the data from mice in DD compared to LD conditions (see Supplementary Information S1 - Differences in DD and LD regimes). In particular, we observed larger *Per2* amplitudes and earlier phases of several genes. Control analysis of our model has lead to explanations of these differences. Since systemic regulation of *Per2* was reported [15], simulated external forcing of *Per2* can reproduce the amplitude increase and phase advances (see Figure 7). Interestingly, almost identical effects can be simulated by changing 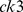, a parameter that directly controls *Per2* expression.

Even though these model changes explained most of the DD-LD differences, the amplitude decrease and phase advance of *Cry1* under LD conditions require other parameter adjustments. Control analysis suggests that an increase of 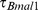 in LD conditions might explain these differences. It seems reasonable that the metabolic conditions in liver are different in DD and LD regimes, and this could lead to modification of the *Bmal1* dynamics. Even though food was available *ad libitum* in our experiments, LD conditions might change food intake patterns affecting in turn Bmal1 expression [53].

### Intracellular feedback loops govern liver clock

Our cell-autonomous model explains the gene expression patterns surprisingly well. The model was designed to represent the DD situation, but relatively small parameter adjustments were sufficient to reproduce also the LD rhythms. The excellent agreement of simulations and experiments supports the findings of [15] that the intrinsic oscillations dominate the circadian clock. Even though our focus was on the core clock genes, the similarity of DD and LD rhythms in 15 additional genes (see Supplementary Information S1 - Fitting of trigonometric functions to gene expression data) suggest that the physiological function of liver is ruled by intracellular regulation.

In the last years, various models of the circadian clock have been developed. Some of them focus on the phases, amplitudes, temperature compensation, and entrainment properties [54]–[57], and others focus on many biochemical details [13], [19]. Here, we developed a different approach: our six-variable model simulates selected genes and regulatory elements, and contains relatively few parameters due to explicit delays, which reflect the protein dynamics. The kinetic terms are straightforward translations of thermodynamic principles and the number of *cis*-regulatory elements has been extracted from literature (Supplementary Information S4 - Clock-controlled elements). The degradation rates have been chosen based on experimental studies and the remaining parameters have been fitted to our gene expression data shown in Figure 1.

The relatively simple structure of our delay-differential equations allows in-depth studies of the underlying design principles: overcritical delays are needed to obtain oscillations, and synergies of activators and inhibitors enhance amplitudes. Our concept of “modulation factors” provides quantitative understanding of combinatorial gene regulation. The high level of agreement between our measured expression profiles and our gene regulatory model suggests that intracellular feedbacks govern the liver clock whereas systemic cues just fine-tune the rhythms.

## Materials and Methods

### Animals and tissue samples

Altogether, 121 C57BL/6JOlaHsd mice were used, and they had free access to food (Harland Tekland 2916) and water. All of the animals were initially kept under a 12 h∶12 h LD cycle (lights on at 07:00; lights off at 19:00) for 3 weeks for their entrainment. Then 60 of these mice were sacrificed by cervical dislocation under LD cycles every 2 h over a 24 h period (at least 4 mice sacrificed per time point). The other 61 mice were put into constant darkness (DD) and were sacrificed after 36 h in DD, under dim red light. The liver of each mouse was excised immediately after their sacrifice, and then snap frozen in liquid nitrogen and stored at −80 C.

The experiments were approved by the Veterinary Administration of the Republic of Slovenia (licence numbers 34401-38/2009/2 and 34401-44/2009/2) and were conducted in agreement with the European Convention for the protection of vertebrate animals used for experimental and other scientific purposes (ETS 123), as well as in agreement with the National Institutes of Health guidelines for work with laboratory animals.

### RNA extraction and cDNA preparation

The liver samples were homogenised, and the total RNA was isolated according to manufacturer instructions (QuickGene RNA tissue kit S, QuickGene 810, FujiFilm LifeScience). The RNA quantity and quality were assessed with NanoDrop and Agilent 2100 Bioanalyzer instruments. DNAse treatment was performed on all of the samples, using DNAse I (Roche Applied Bioscience), according to the manufacturer instructions. The cDNA synthesis was carried out using SuperScript III reverse transcriptase (Invitrogen). Liver RNA (3 mg) was mixed with 20 ml reverse transcriptase master mix, which contained 8 ml 5 first strand buffer, 2 ml 100 mM dithiothreitol, 2 ml 10 mM dNTP mix, 1 ml random primers (Promega 500 ng/ml), 0.75 ml SuperScript III (200 U/ml), 0.75 ml RNAse OUT (Invitrogen), and 5.5 ml RNAse free water, giving a final volume of 40 ml. The reaction mixtures were incubated at 25 C for 5 min, 50 C for 60 min, and 70 C for 10 min.

### Real time qPCR

Intron-spanning primers for the clock and normalisation genes were designed based on the available gene sequences (Supplementary Information S6). The primer specificities and amplification efficiencies were validated empirically with melting curve and standard curve analysis of a six-fold dilution series.

Real-time quantitative PCR was performed in a 384-well format on a LightCycler 480 (Roche Applied Science), using SYBR Green I Master (Roche Applied Science). The PCR reactions consisted of 2.5 ml SYBR Green I Master, 1.15 ml RNAse free water, 0.6 ml 300 nM primer mix and 0.75 ml cDNA, to a total volume of 5 ml. Three technical replicates were performed for each sample. The cycling conditions were: 10 min at 95 C, followed by 40 rounds of 10 s at 95 C, 20 s at 60 C, and 20 s at 72 C. The melting curve analyses for determining the dissociation of the PCR products were performed from 65 C to 95 C.

The Cp values of the expressed genes were transformed into quantities by taking into account the primer efficiencies (Supplementary Information S6). These quantities were then normalised by a normalisation factor, i.e. the geometric mean of the expression of the reference genes *Hmbs*, *Eif2A*, and *Ppib* [23].

### Model simulation

Delay-differential equations with constant delays were implemented in MATLAB and were solved using the dde23 function. Bifurcation analysis was performed using DDE-BIFTOOL v. 2.03

(http://twr.cs.kuleuven.be/research/software/delay/ddebiftool.shtml) implemented in MATLAB.

The values of degradation rates 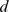 and explicit delays 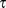 are based on published data (Supplementary Information S4 - Parameter estimation) and the exponents represent the numbers of experimentally verified binding sites are fixed (Supplementary Information S4 - Clock-controlled elements). The remaining parameter values are optimised to reproduce phases, amplitudes, and waveforms of our measured expression profiles. Detailed analysis of the process is described in Supplementary Information S4 - Parameter estimation.

## Supporting Information

Supplementary Information S1 **Experimental data and regression.** Additional information on experimental data analysis is presented in three sections: Fitting of trigonometric functions to gene expression data; Parameters describing the oscillatory gene expression; Differences in DD and LD regimes.
(PDF)

Supplementary Information S2 **Oscillations in a delay-differential equation.** Mathematical details regarding steady states, oscillation onset, and the relation between delay and oscillation period.
(PDF)

Supplementary Information S3 **Theory of combinatorial regulation.** Supporting information on theory of combinatorial regulation used in the main text is provided in three sections: Multiplying transcriptional modulator factors; Synergy of antiphase activators and inhibitors; Long half-lives shift expression peaks.
(PDF)

Supplementary Information S4 **Model design and parameters.** Supporting information explaining determination of clock-controlled elements and parameter estimation is presented in two sections: Clock-controlled elements; Parameter estimation - literature data, sensitivity analysis, and fitting.
(PDF)

Supplementary Information S5 **Control analysis - robustness with respect to model parameters.** Details on control analysis.
(PDF)

Supplementary Information S6 **Primers.** Additional information on RT-PCR and primers used in the study.
(PDF)

Spreadsheet S1 **Control analysis for all parameters.** Spreadsheet shows relative changes in system variables as a percentage of the reference value (at default parameter value). For period length and phases, changes larger than 1% are marked. For amplitudes, we put the limit at 3%. Red and green refer to increasing and decreasing values, respectively.
(XLS)
