# Supplementary material for: The Interplay of cis-Regulatory Elements Rules Circadian Rhythms in Mouse Liver
Source: PLoS One. 2012 Nov 5;7(11):e46835. doi: 10.1371/journal.pone.0046835 (PMC3489864; doi:10.1371/journal.pone.0046835)
Supplement: Supplementary Information S1 — Experimental data and regression. Additional information on experimental data analysis is presented in three sections: Fitting of trigonometric functions to gene expression data; Parameters describing the oscillatory gene expression; Differences in DD and LD regimes. (PDF) [file pone.0046835.s001.pdf]

S1 Experimental data and regression

S1.1 Fitting of trigonometric functions to gene expression data

Experimental data were fitted by trigonometric functions. In addition to the 24 h period we also included 12 h and 8 h harmonics to represent variable waveforms:

$$x_t = a1 \sin\left(\frac{2\pi}{24h}t\right) + a2 \cos\left(\frac{2\pi}{24h}t\right) + b1 \sin\left(\frac{2\pi}{12h}t\right) + b2 \cos\left(\frac{2\pi}{12h}t\right) + c1 \sin\left(\frac{2\pi}{8h}t\right) + c2 \cos\left(\frac{2\pi}{8h}t\right) + d \quad \text{(S1)}$$

Figures S1 and S2 present the fits with the 24 h period together with the 12 h harmonics from reduced Equation (S1).

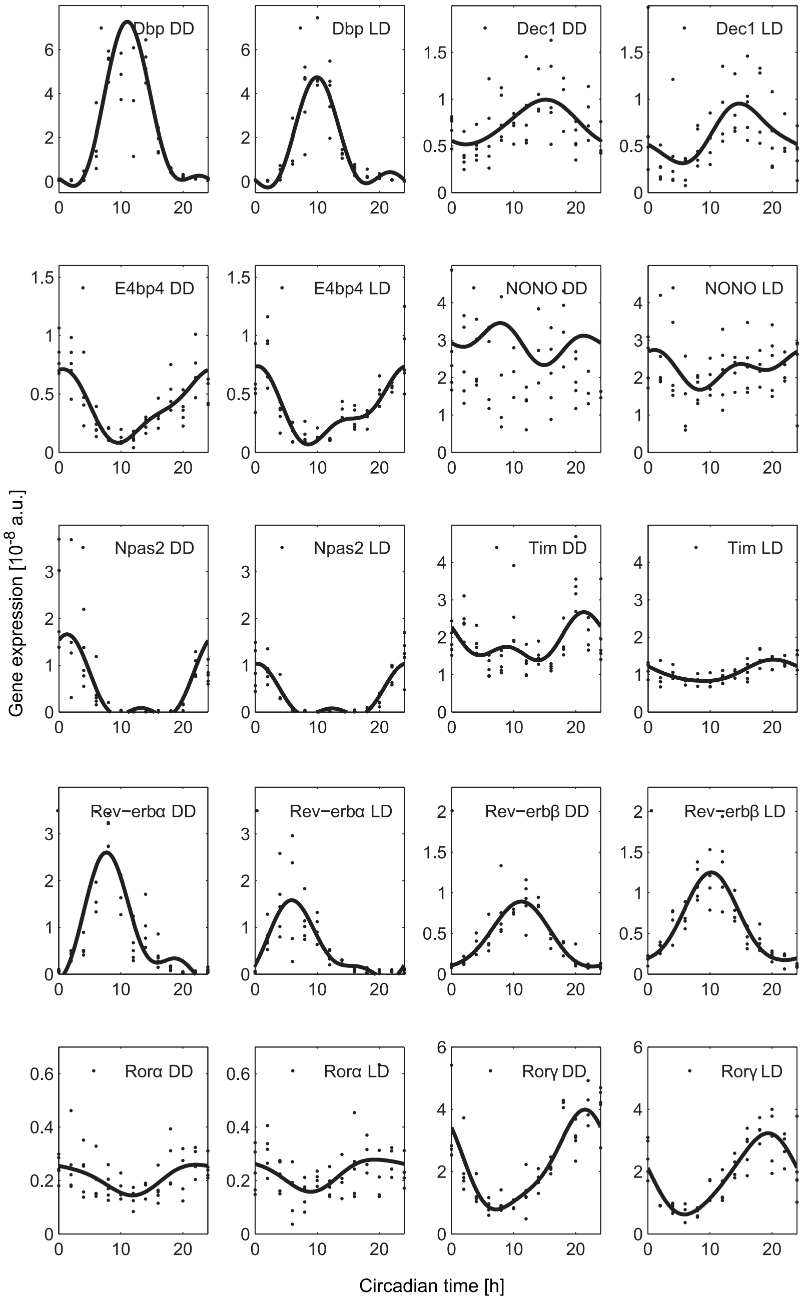

Figure S1: Circadian expression of clock related genes and regression using reduced Equation (S1). The raw data were normalised by a combination of three reference genes as explained in Materials and methods and are represented by the black dots. Parameter values for the fits are shown in Table S2.

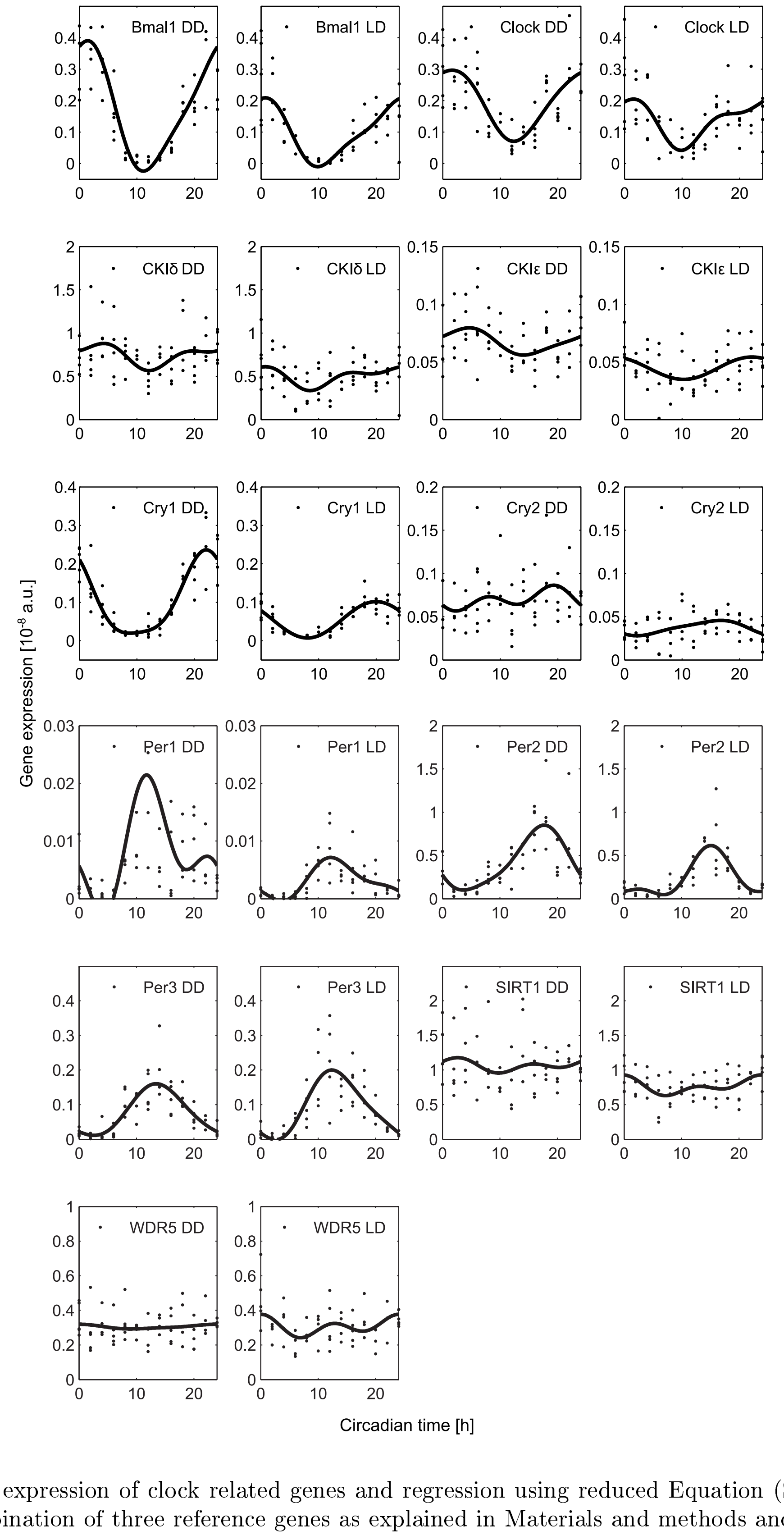

Figure S2: Circadian expression of clock related genes and regression using reduced Equation (S1). The raw data were normalised by a combination of three reference genes as explained in Materials and methods and are represented by the black dots. Parameter values for the fits are shown in Table S2.

Fits and model simulations were normalised, i.e. divided by the mean expression of each gene. All subsequent comparisons in our paper were performed using normalised data. The fits yield the parameter values in Tables S1 - S3.

	**Dark-dark (DD)**			**Light-dark (LD)**		
**Gene**	$a1$	$a2$	d	$a1$	$a2$	d
Bmal1	2.26	19.54	17.58	-1.41	10.02	9.82
Clock	2.44	10.71	19.01	-1.86	6.42	13.50
Cry1	-5.61	9.38	10.56	-4.07	2.40	5.45
Dbp	75.19	-321.21	245.99	103.79	-184.64	148.55
Dec1	-15.84	-17.81	74.53	-24.58	-14.49	62.08
E4bp4	-5.39	28.35	39.03	-6.08	28.16	37.50
Npas2	27.20	78.63	52.68	3.90	51.56	32.30
Per1	-0.28	-0.72	0.84	-0.13	-0.27	0.33
Per2	-35.11	-9.08	42.27	-19.12	-16.18	25.03
Per3	-3.41	-6.42	8.03	-3.14	-8.86	9.50
Rev-erbα	98.79	-69.08	88.21	73.75	-13.54	54.26
Rev-erbβ	9.09	-38.07	44.86	24.34	-47.33	62.54
Rorα	-0.87	5.37	20.78	-4.59	3.69	22.74
Rorγ	-112.83	106.93	219.21	-125.58	25.06	186.95
Tim	-26.92	38.59	186.56	-23.38	16.46	107.60
Cry2	-0.76	-0.19	6.99	-0.62	-0.57	3.71
WDR5	-0.36	1.23	30.52	-1.59	3.50	30.97
CKIδ	3.90	10.48	74.22	-6.33	8.76	49.93
CKIε	0.86	0.65	6.74	-0.44	0.86	4.45
Nono	28.42	7.54	291.99	-14.38	32.45	225.28
Sirt1	0.76	6.17	106.62	-4.30	9.86	77.23

Table S1: Parameter values for fitting the experimental data with Equation (S1) without 12 h and 8 h harmonics. All parameter values are multiplied with 10^{10}.

	Dark-dark (DD)					Light-dark (LD)				
**Gene**	$a1$	$a2$	$b1$	$b2$	d	$a1$	$a2$	$b1$	$b2$	d
Bmal1	2.22	19.45	3.65	0.11	17.53	-1.55	9.77	2.30	1.11	9.68
Clock	2.40	10.87	0.54	-1.09	19.07	-1.97	6.46	2.88	-0.34	13.44
Cry1	-5.54	9.23	-1.90	1.39	10.52	-4.07	2.40	0.02	-0.02	5.45
Dbp	79.78	-341.75	-71.35	112.96	242.67	105.04	-188.86	-100.44	49.21	146.61
Dec1	-15.89	-17.64	1.04	-1.22	74.59	-24.69	-15.40	5.14	5.29	61.61
E4bp4	-5.35	27.54	6.72	4.35	38.70	-6.23	26.45	6.35	10.36	36.63
Npas2	27.77	73.74	23.35	28.34	50.72	3.70	47.69	4.23	24.89	30.34
Per1	-0.26	-0.78	-0.23	0.54	0.82	-0.13	-0.29	-0.05	0.11	0.32
Per2	-35.31	-7.98	-1.58	-6.87	42.69	-19.30	-16.44	11.31	0.15	24.88
Per3	-3.39	-6.55	0.20	0.79	7.99	-3.14	-9.03	-0.86	1.48	9.42
Rev-erbα	98.06	-62.16	-37.43	-39.50	91.01	73.79	-9.43	7.02	-27.98	56.33
Rev-erbβ	9.26	-38.79	-1.06	4.76	44.59	24.42	-48.02	-7.11	5.46	62.21
Rorα	-0.90	5.52	-0.31	-0.90	20.84	-4.60	3.71	1.09	-0.25	22.74
Rorγ	-111.82	104.34	-25.50	20.01	218.32	-125.37	25.44	-14.01	-0.62	187.18
Tim	-26.36	38.88	-33.64	2.53	186.80	-22.93	16.73	-3.25	-2.41	107.99
Cry2	-0.77	-0.08	-0.70	-0.61	7.03	-0.62	-0.54	-0.08	-0.16	3.73
WDR5	-0.36	1.18	0.14	0.31	30.50	-1.63	2.83	0.89	4.27	30.63
CKIδ	3.68	11.47	1.34	-6.50	74.60	-6.43	8.23	5.47	2.75	49.65
CKIε	0.85	0.69	-0.02	-0.24	6.75	-0.44	0.87	0.00	-0.01	4.46
Nono	28.59	9.50	-32.50	-9.53	292.85	-14.82	29.55	22.97	16.00	223.77
Sirt1	0.65	6.12	6.38	-0.57	106.58	-4.36	8.70	1.17	7.48	76.64

Table S2: Parameter values for fitting the experimental data with Equation (S1) without 8 h harmonics. All parameter values are multiplied with 10^{10}.

	Dark-dark (DD)						
	$a1$	$a2$	$b1$	$b2$	$c1$	$c2$	d
Bmal1	2.21	19.90	3.61	0.56	0.20	-3.77	17.77
Clock	2.40	10.74	0.55	-1.22	-0.10	1.06	19.00
Cry1	-5.55	9.20	-1.90	1.37	-0.21	0.34	10.50
Dbp	79.60	-341.88	-70.99	112.69	-10.54	4.43	242.26
Dec1	-15.99	-18.65	1.32	-2.08	-6.94	9.39	73.98
E4bp4	-5.28	27.18	6.62	3.91	3.70	2.34	38.57
Npas2	27.86	73.21	23.23	27.71	4.68	3.59	50.52
Per1	-0.26	-0.73	-0.23	0.59	0.09	-0.53	0.84
Per2	-35.30	-8.87	-1.52	-7.76	-0.21	7.33	42.24
Per3	-3.40	-6.58	0.22	0.76	-0.54	0.39	7.96
Rev-erbα	97.76	-66.02	-36.54	-42.92	-21.12	35.21	88.74
Rev-erbβ	9.23	-38.51	-1.02	5.07	-1.73	-1.97	44.70
Rorα	-0.88	5.51	-0.35	-0.94	1.19	-0.07	20.85
Rorγ	-111.74	101.95	-25.45	17.55	2.78	19.14	217.16
Tim	-25.80	42.38	-34.99	5.27	36.10	-34.71	189.12
Cry2	-0.76	-0.15	-0.70	-0.68	0.20	0.54	7.00
WDR5	-0.36	0.91	0.17	0.05	-0.50	2.28	30.36
CKIδ	3.71	11.23	1.29	-6.78	2.02	1.59	74.51
CKIε	0.85	0.65	-0.03	-0.29	0.33	0.30	6.74
Nono	28.02	7.43	-31.24	-10.83	-35.92	27.45	291.26
Sirt1	0.40	6.02	6.87	-0.34	-15.20	3.35	106.30
	**Light-dark (LD)**						
	$a1$	$a2$	$b1$	$b2$	$c1$	$c2$	d
Bmal1	-1.83	9.65	2.23	1.20	2.57	0.65	9.48
Clock	-1.98	6.19	2.79	-0.55	1.50	1.72	13.33
Cry1	-4.10	2.31	-0.02	-0.07	0.62	0.66	5.40
Dbp	104.30	-189.62	-101.72	48.99	42.77	-2.09	146.13
Dec1	-24.69	-15.96	5.13	4.74	1.35	4.37	61.33
E4bp4	-6.30	26.07	6.24	10.03	4.34	2.34	36.43
Npas2	3.70	46.86	4.23	24.06	1.59	6.54	29.93
Per1	-0.13	-0.28	-0.05	0.11	0.08	-0.07	0.32
Per2	-19.24	-17.31	11.41	-0.77	-1.66	7.50	24.45
Per3	-3.18	-9.02	-0.93	1.52	2.17	-0.59	9.42
Rev-erbα	73.76	-7.98	6.97	-26.50	-1.06	-11.78	57.05
Rev-erbβ	24.33	-47.19	-7.27	6.36	3.65	-7.50	62.61
Rorα	-4.66	3.77	0.99	-0.14	3.14	-1.13	22.77
Rorγ	-125.43	24.45	-14.13	-1.56	5.66	7.12	186.67
Tim	-22.87	16.72	-3.24	-2.47	-0.47	0.15	108.02
Cry2	-0.63	-0.54	-0.09	-0.16	0.42	-0.09	3.73
WDR5	-1.64	2.66	0.86	4.12	1.28	1.15	30.55
CKIδ	-6.55	7.86	5.26	2.47	7.27	1.74	49.45
CKIε	-0.44	0.85	-0.01	-0.02	0.27	0.11	4.45
Nono	-15.11	30.40	22.47	17.06	14.58	-9.63	224.16
Sirt1	-4.40	8.42	1.09	7.23	2.98	1.73	76.50

Table S3: Parameter values for fitting the experimental data with Equation (S1). All parameter values are multiplied with 10^{10}.

S1.2 Parameters describing the oscillatory gene expression

All parameters of peak characterization from Tables S4 and S5 are derived from the normalised fits of the periodic genes. Gene expression of each gene was described by peak phase, amplitude, and width. Differences exist between fits with additional harmonics, so the tables show all fitting versions.

In Tables S4 and S5, 'phase' describes the peak of gene expression; 24 h, 12 h, and 8 h identify the number of harmonics that were used while fitting the experimental data with Equation S1. Maximum (Max) and minimum (Min) take the largest and smallest value of the fit and thus represent the amplitude of the oscillations. Peak width at half of the maximum (PWHM) describes the width of the peak in hours for the fit with 12 h harmonics. Parameter termed '% of pure sin' shows the fraction of the first two fitting parameters $\frac{a1^2+a2^2}{a1^2+a2^2+b1^2+b2^2+c1^2+c2^2}$ in the fit with included 12 h and 8 h harmonics and thus represents the fraction of pure sine wave with a 24 h period.

**Gene**	**Phase (24 h)**	**Phase (12 h)**	**Phase (8 h)**	**Max (12 h)**	**Min (12 h)**	**Max (8 h)**	**Min (8 h)**	**PWHM (12 h)**	**% of pure sin**
Bmal1	0.4	1.4	2.6	2.21	-0.14	2.25	-0.07	11.1	0.94
Clock	0.9	1.6	0.8	1.55	0.37	1.57	0.31	12.9	0.98
Cry1	21.9	22.1	22.2	2.24	0.19	2.26	0.18	9.0	0.95
Dbp	11.1	11.0	11.1	3.01	-0.08	2.97	-0.13	7.9	0.87
Dec1	14.8	15.2	15.2	1.33	0.69	1.51	0.53	11.3	0.81
E4bp4	23.3	0.6	0.9	1.84	0.21	1.92	0.31	10.9	0.91
Npas2	1.3	1.3	1.3	3.25	-0.16	3.35	-0.04	7.5	0.82
Per1	13.4	11.8	11.8	2.63	-0.33	3.22	-0.14	7.9	0.47
Per2	17.0	17.7	16.8	2	0.24	2.13	0.05	9.8	0.92
Per3	13.9	13.4	13.9	2.01	0.14	2.08	0.05	10.7	0.98
Rev-erbα	8.3	7.7	7.5	2.87	-0.13	3.41	-0.13	7.8	0.74
Rev-erbβ	11.1	11.3	11.7	2.01	0.21	2.03	0.21	10.1	0.98
Rorα	23.4	22.1	1.3	1.24	0.69	1.24	0.67	13.9	0.93
Rorγ	20.9	21.5	22.2	1.82	0.36	1.79	0.39	9.7	0.95
Tim	21.7	21.3	19.7	1.43	0.74	1.57	0.48	7.2	0.4
Cry2	17.1	19.3	17.7	1.23	0.81	1.24	0.77	15.8	0.32
WDR5	22.9	23.9	23.7	1.05	0.96	1.11	0.89	9.8	0.15
CKIδ	1.4	4.3	3.1	1.18	0.76	1.16	0.73	15.7	0.72
CKIε	3.5	4.6	2.0	1.18	0.83	1.17	0.77	11.5	0.8
Nono	5.0	7.8	7.1	1.18	0.8	1.33	0.81	16.4	0.21
Sirt1	0.5	2.7	14.5	1.11	0.9	1.16	0.75	15.9	0.11

Table S4: Peak characterisation of gene expression data in DD. By using Equation (S1) with different number of harmonics, we get slightly different fits leading to variations in peak phases and amplitudes.

Gene	Phase (24 h)	Phase (12 h)	Phase (8 h)	Max (12 h)	Min (12 h)	Max (8 h)	Min (8 h)	PWHM (12 h)	% of pure sin
Bmal1	23.5	0.7	1.2	2.14	-0.1	2.4	0.03	11.3	0.88
Clock	22.9	1.6	1.2	1.52	0.31	1.65	0.35	15.2	0.76
Cry1	20.0	20.0	18.1	1.86	0.13	1.88	0.14	11.9	0.96
Dbp	10.0	9.9	10.0	3.25	-0.18	3.56	-0.02	7.4	0.76
Dec1	16.0	14.7	15.5	1.55	0.51	1.6	0.45	10.6	0.93
E4bp4	23.2	0.4	0.7	2	0.18	2.1	0.26	8.8	0.81
Npas2	0.3	0.3	0.3	3.38	-0.21	3.57	0.02	7.3	0.77
Per1	13.7	12.2	11.5	2.26	-0.1	2.49	-0.04	9.7	0.79
Per2	15.3	15.1	15.4	2.48	0.19	2.83	0.08	7.7	0.78
Per3	13.3	12.4	11.2	2.13	-0.01	2.25	0.11	10.9	0.92
Rev-erbα	6.7	5.9	5.3	2.82	-0.18	2.86	-0.1	8.2	0.86
Rev-erbβ	10.2	10.2	10.8	2.02	0.28	2.11	0.19	9.6	0.95
Rorα	20.6	19.2	18.6	1.22	0.69	1.37	0.61	14.3	0.75
Rorγ	18.8	19.4	18.7	1.73	0.33	1.72	0.3	11.2	0.98
Tim	20.3	20.2	20.3	1.3	0.77	1.3	0.77	9.9	0.98
Cry2	15.2	16.8	17.9	1.23	0.75	1.32	0.75	12.9	0.76
WDR5	22.4	0.0	0.5	1.23	0.79	1.27	0.77	16.2	0.32
CKIδ	21.6	0.8	1.4	1.23	0.68	1.35	0.65	15.9	0.54
CKIε	22.2	22.1	0.6	1.22	0.78	1.21	0.77	11.9	0.91
Nono	22.4	1.1	1.9	1.22	0.75	1.27	0.7	15.9	0.51
Sirt1	22.4	23.8	0.4	1.21	0.82	1.23	0.81	7.8	0.58

Table S5: Peak characterisation of gene expression data in LD. By using Equation (S1) with different number of harmonics, we get slightly different fits leading to variations in peak phases and amplitudes.

S1.3 Differences in DD and LD regimes

To evaluate the differences in gene expression in DD and LD regimes, we performed bootstrapping on the original data. Each time, half of the data points were randomly chosen and all parameters from Tables S4 and S5 were re-calculated. After 100 repetitions, results are plotted as box-plots. Figure S3 shows results for the genes included in our model.

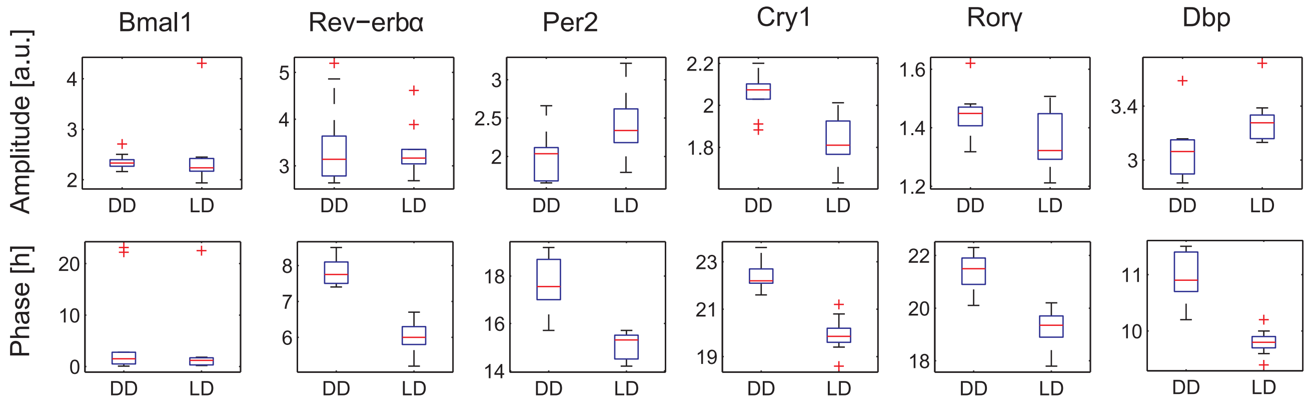

Figure S3: Differences in amplitude and phase of oscillation in dark-dark (DD) and light-dark (LD) conditions for the six genes included in our model.
