# Supplementary material for: The Interplay of cis-Regulatory Elements Rules Circadian Rhythms in Mouse Liver
Source: PLoS One. 2012 Nov 5;7(11):e46835. doi: 10.1371/journal.pone.0046835 (PMC3489864; doi:10.1371/journal.pone.0046835)
Supplement: Supplementary Information S2 — Oscillations in a delay-differential equation. Mathematical details regarding steady states, oscillation onset, and the relation between delay and oscillation period. (PDF) [file pone.0046835.s002.pdf]

S2 Oscillations in a delay-differential equation

We modelled the self-inhibition of a gene using a one-variable delay differential equation (DDE). Nonlinearities and sufficiently long delays generated self-sustained oscillations. Here we provide mathematical details regarding steady states, oscillation onset (Hopf bifurcation) and the relation between delay and oscillation period.

We consider a one-variable delay-differential equation in the form of

$$\frac{\mathrm{d}x}{\mathrm{d}t} = g\left(x\left(t-\tau\right)\right) - d \cdot x, \tag{S2}$$

where the variable x describes the abundance of a clock gene (e.g. *Per2*). The nonlinear function $g\left(\right)$ describes the self-inhibition of the gene, and $d > 0$ is a degradation rate of the mRNA. For example, we might assume a nonlinear negative feedback in the form of

$$g\left(x\right) = \left(\frac{a}{b+x}\right)^2, \tag{S3}$$

as described in the main text. The delay τ corresponds to the time needed for translation, post-translational modifications, complex formation, and translocation of the protein.

Steady state

A steady state of Equation (S2) satisfies $\frac{\mathrm{d}x}{\mathrm{d}t} = 0$ and is given by the nonlinear equation

$$g\left(x\right) - d \cdot x = 0,$$

which, for our specific choice of the function $g\left(\right)$, is equivalent to

$$a^2 - d \cdot \left(b+x\right)^2 x = 0. \tag{S4}$$

This is a nonlinear equation, whose solutions determine the steady states of Equation (S2). Generally, for more complicated kinetics $g\left(\right)$, the steady state equation can be solved only numerically.

Linear stability analysis

In order to determine the stability of the previously found steady state, we consider the fate of small perturbations. Suppose that we have the steady state given by $x = x_0$. Now we introduce a small perturbation $y\left(t\right)$:

$$x\left(t\right) = x_0 + y\left(t\right). \tag{S5}$$

In order to determine the stability of x_0, we need to understand whether the deviation $y\left(t\right)$ would grow or decay in time. The dynamics of $y\left(t\right)$ is given by our original DDE:

$$\frac{\mathrm{d}y}{\mathrm{d}t} = \frac{\mathrm{d}x}{\mathrm{d}t} = g\left(x\left(t-\tau\right)\right) - d \cdot x,$$

where the first equality follows directly from the definition of y given by Equation (S5). Now we exploit that $y\left(t\right)$ is small and approximate the nonlinear function $g\left(\right)$ by a linear function centered at $g\left(x_0\right)$ with the slope J:

$$\frac{\mathrm{d}y}{\mathrm{d}t} \approx g\left(x_0\right) + Jy\left(t-\tau\right) - d \cdot x_0 - d \cdot y\left(t\right).$$

For a steady state, $g\left(x_0\right) - d \cdot x_0 = 0$ holds and we obtain

$$\frac{\mathrm{d}y}{\mathrm{d}t} = Jy\left(y-\tau\right) - d \cdot y\left(t\right). \tag{S6}$$

This is a DDE for the unknown function $y(t)$. This equation is linear and can be solved by an exponential ansatz:

$$y(t) = y_0 \cdot \mathrm{e}^{\lambda t}$$

with an unknown complex number λ. Substituted in Equation (S6), this leads to

$$y_0 \cdot \lambda \cdot \mathrm{e}^{\lambda t} = J \cdot y_0 \cdot \mathrm{e}^{\lambda(t-\tau)} - d \cdot y_0 \cdot \mathrm{e}^{\lambda t}.$$

Dividing by $y_0 \cdot \mathrm{e}^{\lambda t}$ results in a transcendental characteristic equation for λ:

$$\lambda = J \cdot \mathrm{e}^{-\lambda\tau} - d. \tag{S7}$$

Reversing the chain of thoughts, as soon as we have found λ which solves Equation (S7), the function $y(t) = y_0 \cdot \mathrm{e}^{\lambda t}$ would be a solution to Equation (S6). The time dependence of $y_0 \cdot \mathrm{e}^{\lambda t}$ depends on the sign of the real part of λ. If $\mathrm{Re}\,\lambda < 0$, the function $y(t)$ will decay in the course of time, corresponding to a stable steady state x_0. If $\mathrm{Re}\,\lambda > 0$, the function $y(t)$ grows, i.e. the system departs from steady state x_0 and the steady state is unstable.

Summarizing, given a steady state x_0, we have to solve Equation (S7) for the unknown λ, whose real part would determine the stability of x_0. Note that Equation (S7) depends on the steady state itself through J, on the value of the time delay τ, and on the degradation rate d. Thus we expect that the stability of the steady state can be changed by tuning one of those parameters. In general, the transcendental equation (S7) can be solved only numerically. Below we show that near the oscillation onset at $\mathrm{Re}\,\lambda = 0$, Equation (S7) can be reduced to a simpler one which can be solved analytically.

Oscillation onset (Hopf bifurcation)

Here, we are interested in a special situation where the complex number λ has a zero real part. This corresponds to parameters where the stability of steady state changes: if we vary a parameter slightly, the real part of λ would become non-zero and the steady state would either loose or gain stability.

We introduce the real and the imaginary part of λ by $\lambda = \mu + \mathrm{i}\omega$, which transforms the complex Equation (S7) into two real equations as follows:

$$\begin{aligned} \mu &= J\mathrm{e}^{-\mu\tau}\cos(\omega\tau) - d, \\ \omega &= -J\mathrm{e}^{-\mu\tau}\sin(\omega\tau). \end{aligned}$$

The situation $\mathrm{Re}\,\lambda = 0$ is associated with a change of stability of the steady state. For non-zero ω, a periodic solution (a "limit cycle") emerges from the steady state, which at the same time looses its stability. This scenario is known as a Hopf bifurcation. The condition $\mu = 0$ simplifies the above equations to

$$\begin{aligned} J\cos(\omega\tau) - d &= 0, \\ -J\sin(\omega\tau) &= \omega. \end{aligned}$$

Using the trigonometric identity $\cos^2(\omega\tau) + \sin^2(\omega\tau) = 1$, we obtain

$$J^2 = d^2 + \omega^2.$$

From these formulae, we can derive an expression for the critical delay τ at the Hopf bifurcation:

$$\cos(\omega\tau) = \frac{d}{J} \quad \Rightarrow \quad \tau = \frac{\arccos\frac{d}{J}}{\omega} = \frac{\arccos\frac{d}{J}}{\sqrt{J^2 - d^2}}.$$

This expression implies that the critical delay τ depends only on the degradation rate d and the slope J of the function $g()$ at the steady state x_0. For self-inhibition, the slope J is negative and, hence, we can find solution for $-1 < \frac{d}{J} < 0$. Note that J has to be larger than d owing to the relation $J^2 = d^2 + \omega^2$. This implies that $\arccos \frac{d}{J}$ is monotonically increasing from $\frac{\pi}{2}$ (corresponding to $\frac{d}{J} = 0$) to π (for $\frac{d}{J} = -1$). Consequently, the critical delay τ is in the range

$$\frac{\pi}{2\omega} < \tau < \frac{\pi}{\omega}.$$

Since the period T of oscillation close to a Hopf bifurcation is approximately given by $T = \frac{2\pi}{\omega}$, we obtain the following inequality for the critical delay τ and the period T of the emerging oscillations:

$$\frac{T}{4} < \tau < \frac{T}{2}.$$

This is a central result of our model analysis: the delay of the negative feedback loop is in the range from a quarter to a half of the expected oscillation period. For a circadian period of $T = 24\,\mathrm{h}$, a delay of at least 6 h (and of at most 12 h) is required to get self-sustained oscillations. Furthermore, the equation

$$\tau = \frac{T}{2\pi} \arccos \frac{d}{J}$$

indicates that the critical delay and the oscillation period are linearly proportional to each other. From the relation $J^2 = d^2 + \omega^2$ we deduce that for a given degradation rate d and period $T = \frac{2\pi}{\omega}$, the slope of J has to exceed some threshold in order to get oscillations. This implies that a certain strength of a negative feedback is required. For example, inserting $\omega = \frac{2\pi}{24}\,\mathrm{h}^{-1}$ and $d = 0.25\,\mathrm{h}^{-1}$ leads to a slope $J = -0.57$, which in turn constraints the model parameters of the inhibition kinetics.
