# Supplementary material for: The Interplay of cis-Regulatory Elements Rules Circadian Rhythms in Mouse Liver
Source: PLoS One. 2012 Nov 5;7(11):e46835. doi: 10.1371/journal.pone.0046835 (PMC3489864; doi:10.1371/journal.pone.0046835)
Supplement: Supplementary Information S3 — Theory of combinatorial regulation. Supporting information on theory of combinatorial regulation used in the main text is provided in three sections: Multiplying transcriptional modulator factors; Synergy of antiphase activators and inhibitors; Long half-lives shift expression peaks. (PDF) [file pone.0046835.s003.pdf]

S3 Theory of combinatorial regulation

S3.1 Multiplying transcriptional modulator factors

Our six-variable model of the gene-regulatory network is based on multiple transcriptional regulators. Basic thermodynamics principles (Bintu *et al* (2005)) lead to products of oscillating terms (see Equations (4)-(9)). Figure 6 shows that these products dictate the phases of the corresponding target genes. Several modulation terms resemble trigonometric functions of time and, consequently, we describe in this supplement how the phase and the wave-form of products of sin-functions are determined.

We consider the multiplication of two phase-shifted transcriptional regulators:

$$M(A,\alpha) = (1+\sin(\omega t)) \cdot (1 + A\sin(\omega t + \alpha)). \tag{S8}$$

We assume for simplicity that both regulators oscillate around a basal level of 1 with a period of $\tau = 24\,\mathrm{h}$ corresponding to $\omega = \frac{2\pi}{24}\mathrm{h}^{-1}$. The second sin-term has an amplitude A and is phase-shifted by α. The phase shift of the *Bmal1* modulator and *Per2* modulator in Figure 6B is for example about $\alpha = 2\pi\frac{9}{24}$. Multiplying the brackets in Equation (S8) leads to

$$M(A,\alpha) = 1 + \sin(\omega t) + A\sin(\omega t + \alpha) + A\sin(\omega t) \cdot \sin(\omega t + \alpha).$$

Using trigonometric identities such as

$$\sin(\omega t + \alpha) = \cos\alpha \cdot \sin(\omega t) + \sin\alpha \cdot \cos(\omega t),$$

we obtain

$$M(A,\alpha) = 1 + \frac{1}{2}\cos\alpha + (1 + A\cos\alpha)\cdot\sin(\omega t) + A\sin\alpha\cdot\cos(\omega t) - \frac{A}{2}\cos(2\omega t + \alpha).$$

Thus we have two constant terms, a superposition of sin- and cos-terms, and a term with the double frequency (or, equivalently, half period). The generation of such "harmonics" (i.e. 12 h rhythms) is discussed elsewhere in detail (Westermark, private communication). The amplitude of the 24 h rhythm depends on A and the phase difference α and can be in the range of 0 ("annihilation" of both factors, e.g. for $A = 1$ and $\alpha = \pi$) up to $1 + A$. Of course, in phase oscillators ($\alpha = 0$) enhance the amplitude.

Now we focus on the phase of the 24 h rhythm. For small A, the sin-term dominates and second modulator has only minor effects. This implies, for example, that the low-amplitude D-box modulation in Figure 6C has only a slight effect on the phase of *Cry1*. For $A = 1$ and $\alpha = \frac{\pi}{2}$, the sin- and the cos-terms are equal and the resulting peak phase of the function $M(A,\alpha)$ is right in the middle at $\frac{\pi}{4}$. This implies a kind of phase averaging. Indeed, the products of *Per2* and *Bmal1* modulators in Figure 6B and of E-box and RRE modulators in Figure 6C lead to a phase in between phases of the individual modulators.

In summary, large amplitude modulators have the most pronounced effects on the phase of the product and comparable amplitudes lead to phases in between the peak phases of the factors. Out of phase modulators can cancel each other and can lead to harmonics (Westermark, private communication).

S3.2 Synergy of antiphase activators and inhibitors

As discussed in the main text, our experimental data (Supplementary Information S1) show that many activator-inhibitor pairs (*Bmal1-Dec*, *Dbp-E4bp4*, *Ror*γ*-Rev-erb*α) oscillate antiphasic to each other. As stated earlier (Mitsui *et al* (2001); Ueda *et al* (2005)), this design principle may enhance the clock amplitude. Since these activator-inhibitor pairs bind typically the same sites, their action can be modelled by a sum of oscillatory functions (Bintu *et al* (2005)). The total amplitude A_{tot} can be written as a sum of positive and negative terms representing activator and inhibitor, respectively:

$$\begin{aligned} A_{\mathrm{tot}} &= A_{\mathrm{act}} \cdot \sin(\omega t) - A_{\mathrm{inh}} \cdot \sin(\omega t + \pi) = \\ &= (A_{\mathrm{act}} + A_{\mathrm{inh}}) \cdot \sin(\omega t)\,. \end{aligned}$$

This example illustrates that antiphase inhibitors simply increase the amplitude and keep the phase unchanged. Model parameters in our production terms such as $ak1$ can scale the effective amplitudes of transcriptional regulators. Consequently, by fitting these parameters to the experimental data, we can implicitly include antiphasic counteracting regulators. In this way, the number of genes was reduced successfully.

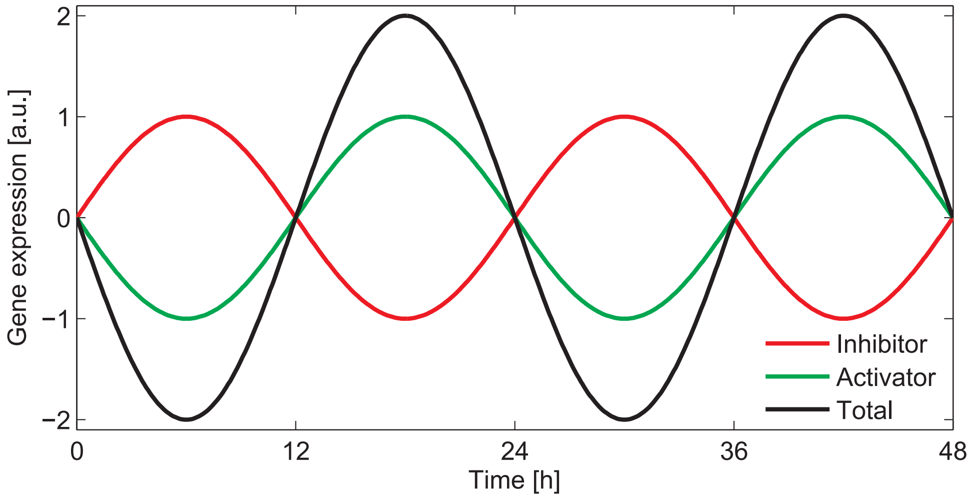

Figure S4: Expression of activator, inhibitor, and their combined effect. The simulated gene expression through a certain CCE (represented by a combined action of activator and inhibitor) is a sine wave with the same phase as the activator, but with doubled amplitude if the amplitudes of activator and inhibitor are the same ($A_{inh} = A_{act} = 1$).

S3.3 Long half-lives shift expression peaks

The thick lines in Figure S5A,B,C represent the time courses of mRNAs of clock genes. These curves are somewhat delayed with respect to their corresponding production terms (dashed line) and have smaller relative amplitudes. These phase shifts and amplitude reductions are closely related to the mRNA half-lives as discussed earlier (Dibner *et al* (2009); Rey *et al* (2011); Suter *et al* (2011); Relogio *et al* (2011)).

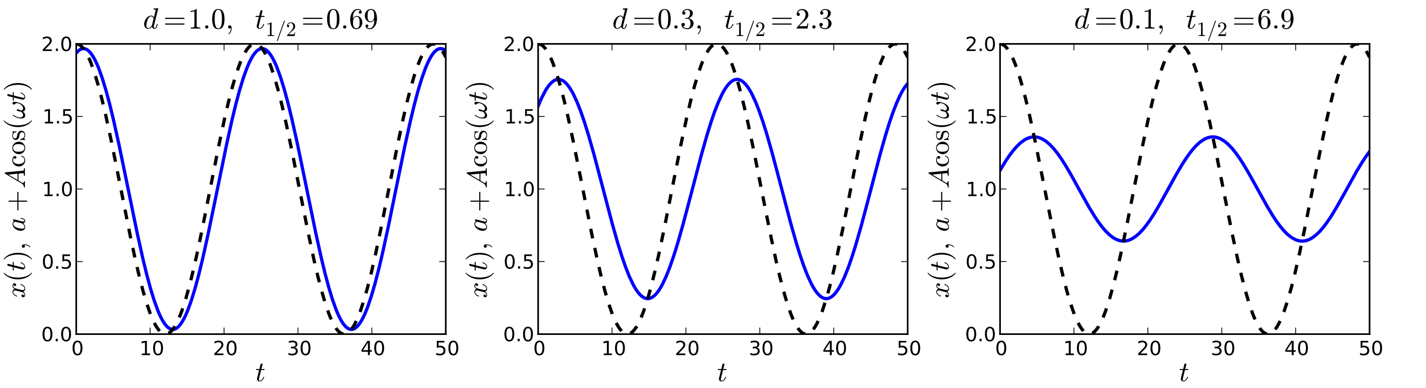

Figure S5: Examples of the solutions $x(t)$ of Equation (S9) (normalised by their mean value a/d) for different degradation rates d and corresponding half-lives $t_{1/2}$. The periodic production $a + A\cos\omega t$ is plotted as dashed lines. For these illustrations, we have chosen $A = 1$, $a = 1$.

We can derive the corresponding dependencies with the help of the following simple equation:

$$\frac{\mathrm{d}x}{\mathrm{d}t} = a + A\cos(\omega t) - d \cdot x. \tag{S9}$$

Here, x denotes the mRNA concentration driven by a basal transcription a and a periodic production term of amplitude A. The parameter d is the degradation rate closely related to the half-life of the mRNA: $t_{1/2} = \frac{\ln 2}{d}$. We assume a circadian period of 24 h leading to $\omega = \frac{2\pi}{T} \approx 0.26\,\mathrm{h}^{-1}$. Equation (S9) can be solved analytically and its asymptotic solution is given by

$$x(t) = \frac{a}{d} + \frac{A}{d^2+\omega^2}\left(d\cos(\omega t) + \omega\sin(\omega t)\right). \tag{S10}$$

This expression has important implications. For large degradation rates ($d \gg \omega$), the cos-term dominates and thus the mRNA is nearly in phase with the production term. For small degradation rates, i.e. large half-lives, the mRNA oscillation follows the sin-term and can be delayed by up to 6 h.

Figure S5 shows some typical solutions of Equation (S9) for different values of the degradation rate d. The left panel of Figure S6 illustrates the phase shift as a function of the half-life. For a typical mRNA half-life of a few hours (Schwanhausser *et al* (2011)), the delays are almost proportional to the half-life. The degradation rate d also affects the relative amplitude of the oscillations. For slow degradation ($d \ll \omega$), the relative amplitude decays with d, i.e. long half-lives imply small relative amplitudes. These results are illustrated in the right panel of Figure S6, which shows phase delay and relative amplitudes as a function of the half-life $t_{1/2}$.

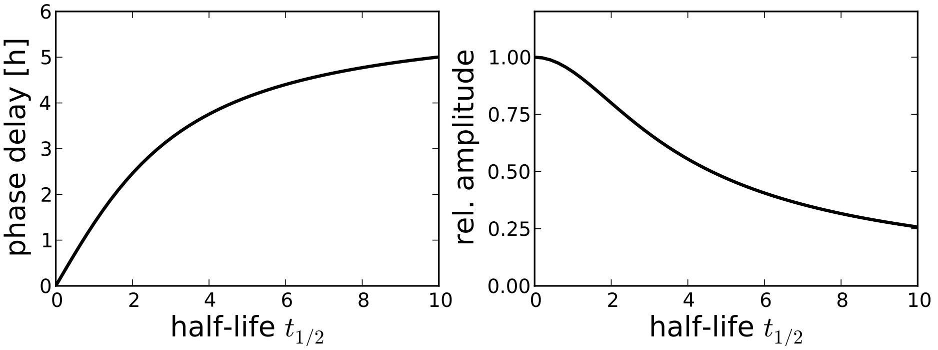

Figure S6: Left panel: phase delay in hours between the driving force $\cos(\omega t)$ and the solution $x(t)$ in Equation (S9) in dependence on the half-life time $t_{1/2}$. Right panel: dependence of the relative (i.e. normalised by the mean value a/d) amplitude of the solution $x(t)$ of Equation (S9) on the half-life time $t_{1/2}$.

References

Bintu L, Buchler NE, Garcia HG, Gerland U, Hwa T, Kondev J, Phillips R (2005) Transcriptional regulation by the numbers: models. *Curr Opin Genet Dev* **15**: 116–124

Dibner C, Sage D, Unser M, Bauer C, d'Eysmond T, Naef F, Schibler U (2009) Circadian gene expression is resilient to large fluctuations in overall transcription rates. *EMBO J* **28**: 123–134

Mitsui S, Yamaguchi S, Matsuo T, Ishida Y, Okamura H (2001) Antagonistic role of E4BP4 and PAR proteins in the circadian oscillatory mechanism. *Genes Dev* **15**: 995–1006

Relogio A, Westermark PO, Wallach T, Schellenberg K, Kramer A, Herzel H (2011) Tuning the mammalian circadian clock: robust synergy of two loops. *PLoS Comput Biol* **7**: e1002309

Rey G, Cesbron F, Rougemont J, Reinke H, Brunner M, Naef F (2011) Genome-wide and phase-specific DNA-binding rhythms of BMAL1 control circadian output functions in mouse liver. *PLoS Biol* **9**: e1000595

Schwanhausser B, Busse D, Li N, Dittmar G, Schuchhardt J, Wolf J, Chen W, Selbach M (2011) Global quantification of mammalian gene expression control. *Nature* **473**: 337–342

Suter DM, Molina N, Gatfield D, Schneider K, Schibler U, Naef F (2011) Mammalian genes are transcribed with widely different bursting kinetics. *Science* **332**: 472–474

Ueda HR, Hayashi S, Chen W, Sano M, Machida M, Shigeyoshi Y, Iino M, Hashimoto S (2005) System-level identification of transcriptional circuits underlying mammalian circadian clocks. *Nat Genet* **37**: 187–192
