# Supplementary material for: The Interplay of cis-Regulatory Elements Rules Circadian Rhythms in Mouse Liver
Source: PLoS One. 2012 Nov 5;7(11):e46835. doi: 10.1371/journal.pone.0046835 (PMC3489864; doi:10.1371/journal.pone.0046835)
Supplement: Supplementary Information S4 — Model design and parameters. Supporting information explaining determination of clock-controlled elements and parameter estimation is presented in two sections: Clock-controlled elements; Parameter estimation - literature data, sensitivity analysis, and fitting. (PDF) [file pone.0046835.s004.pdf]

S4 Model design and parameters

S4.1 Clock-controlled elements

Since our model is based on cis-regulatory regions of clock genes, the determination of the number of clock-controlled elements (CCEs) for each gene was essential. We have taken experimentally validated CCEs as a basis for our model.

Table S6 shows the numbers of clock-controlled elements as used in our model of the circadian clock. For some genes, additional CCEs were predicted but were not tested experimentally.

	**E/E'-box**	**RRE**	**D-box**
**Bmal1**		2 (Preitner *et al* (2002); Yamamoto *et al* (2004))	
**Rev-erbα**	3 (Preitner *et al* (2002); Ueda *et al* (2005); Rey *et al* (2011))		1 (Ueda *et al* (2005))
**Per2**	2 (Ueda *et al* (2005); Ogawa *et al* (2011); Rey *et al* (2011))		1 (Ueda *et al* (2005); Yamajuku *et al* (2010))
**Cry1**	2 (Ukai-Tadenuma *et al* (2011); Rey *et al* (2011))	2 (Ukai-Tadenuma *et al* (2011))	1 (Ukai-Tadenuma *et al* (2011))
**Rorγ**	1 (Ueda *et al* (2005); Rey *et al* (2011))	1 (Ueda *et al* (2005))	
**Dbp**	3 (Ripperger and Schibler (2006); Stratmann *et al* (2010); Rey *et al* (2011))		

Table S6: Number of experimentally verified clock-controlled elements considered in our model.

S4.2 Parameter estimation - literature data, sensitivity analysis, and fitting

Construction of the six-variable model began with the *Bmal1* - *Rev-erba* - *Per2* model by adding *Cry1*, *Rorγ*, and *Dbp* driven solely by *Bmal1*. Degradation rates were chosen as described in the table below, other parameters were initialised with the values of 1.

The second step was the addition of *Per2* inhibition. The term $\left(\frac{c}{ck+[Per2]_{(t-\tau_{Per2})}}\right)^n$ was added to the three new mRNAs (n represents the number of E-boxes of specific gene). Parameter values were adjusted to get optimal phases and amplitudes.

The next step in the model building was the incorporation of *Rev-erba* inhibition of *Cry1* and *Rorγ* by adding the term $\left(\frac{a}{ak+[Rev-erb\alpha]_{(t-\tau_{Rev-erb\alpha})}}\right)^n$, and *Dbp* activation of *Rev-erba*, *Per2*, and *Cry1* by adding $\left(\frac{f+fv[Dbp]_{(t-\tau_{Dbp})}}{fk+[Dbp]_{(t-\tau_{Dbp})}}\right)$ to the production term. The terms were added step by step and parameters were fitted according to their effects noted in the table below.

For the six genes, regulation through combined effect of *Per2* and *Bmal1* on E-boxes could reproduce correct phases, since the interplay of inhibition and activation can create a phase between peak of activation *via Bmal1* and anti-inhibition *via Per2* (Supplementary Information S3.1).

Experimentally determined ranges for explicit delays τ were taken from the time difference between peak of mRNA and protein expression from the same experimental setup (Preitner *et al*, 2002; Lee *et al*, 2001; Hamilton and Kay, 2008). These ranges constricted explicit delay values in our model. Degradation rate ranges were determined by using data from Sharova *et al* (2009), Friedel *et al* (2009), and Suter *et al* (2011). However, some inconsistencies can be observed; in the table below we provide a discussion of the topic. Overall, it has to be taken into account that half-lives measured from Sharova *et al* (2009); Friedel *et al* (2009); Suter *et al* (2011) are from different cell types and thus large differences are

observed.

Symbol	Value	Unit	Description	Parameter notes
τ_{Bmal1}	4	h	Delay of *Bmal1*	The value of 4 h was determined as a compromise between approximately 8 h reported in Preitner *et al* (2002) and almost 0 h in Lee *et al* (2001). Rey *et al* (2011) show that BMAL1 binding has a peak at CT 6, which can be regarded as a conformation of our parameter choice. Increasing τ_{Bmal1} up to 5.2 h increases the period length and amplitudes of all mRNAs. Beyond 5.2 h, the period length shortens to about 18 h and phases and amplitudes of all genes change. τ_{Bmal1} has the strongest effect on *Cry1* and *Rorγ* phases and a non-monotonic effect on *Per2* amplitude.
$\tau_{Rev-erb\alpha}$	1.2	h	Delay of *Rev-erbα*	A value of approximately 2 h was found in Preitner *et al* (2002). This value was adjusted to get the best fit. Increasing $\tau_{Rev-erb\alpha}$ increases the period length and amplitudes of all genes except *Per2* and *Rorγ*.
τ_{Per2}	8.37	h	Delay of *Per2*	The value of 8 h was taken from Lee *et al* (2001) and was adjusted to get the correct phases of model components. Increasing τ_{Per2} increases the period length and amplitudes of all genes considerably. It also has a strong and complex effect on all phases, especially for *Per2*, *Rorγ*, and *Cry1*. For large values ($\tau_{Per2} > 12.5$ h) harmonics can be observed. For values below 7 h, the period length jumps to about 14 h and large changes in amplitudes and phases of all genes are observed. For values below 5.8 h, another change regarding phase length (jumps to about 18 h), amplitudes, and phases of all genes occurs.
τ_{Dbp}	1.5	h	Delay of *Dbp*	Range of 0 - 2 h was reported in Hamilton and Kay (2008); 1.5 h was chosen to get the best fit. Increasing τ_{Dbp} has almost no effect on period length and increases amplitudes of all genes. It has an effect on phase of *Per2*, but also on phases of *Cry1*, *Dbp*, and *Rorγ* , which could also be a network effect through changes in *Per2*.
d_{Bmal1}	0.35	h^{-1}	Degradation rate of *Bmal1* mRNA	The value was chosen in an experimentally determined range of 0.17 - 0.60 h^{-1} (Sharova *et al* (2009); Friedel *et al* (2009); Suter *et al* (2011)). A value of 0.35 h^{-1} was chosen to get the best fit. Increasing d_{Bmal1} increases period length and amplitudes of all genes except *Cry1*. It has the strongest effect on phase differences of *Cry1-Bmal1* and *Rorγ-Bmal1*.

Symbol	Value	Unit	Description	Parameter notes
$d_{Rev-erb\alpha}$	0.60	h^{-1}	Degradation rate of *Rev-erba* mRNA	The initial value was chosen in an experimentally determined range of 0.19 - 0.29 h^{-1} (Sharova *et al* (2009); Friedel *et al* (2009)). The value outside this range was determined to enable the correct phase. This decision is supported by RRE discovery in *Rev-erba* regulatory region (Yamamoto *et al* (2004)), which could explain larger degradation rate (auto-inhibition of *Rev-erba* could cause increase in the effective degradation rate). Increasing $d_{Rev-erb\alpha}$ decreases period length and increases normalised amplitudes of all genes except *Dbp* and *Rev-erba*. It affects mainly phase differences of *Dbp-Bmal1* and *Rev-erba-Bmal1*.
d_{Per2}	0.32	h^{-1}	Degradation rate of *Per2* mRNA	The experimentally determined range is 0.24 - 0.80 h^{-1} (Sharova *et al* (2009); Friedel *et al* (2009)). The value of the parameter was adjusted to get the the correct phase of *Per2*. Increasing d_{Per2} decreases period length and increases normalised amplitudes of all genes. It affects almost all phase differences. Below the value of 0.28 h^{-1}, the period length falls to about 16 h and phases and amplitudes of all genes change.
d_{Cry1}	0.30	h^{-1}	Degradation rate of *Cry1* mRNA	The experimentally determined range is 0.16 - 0.18 h^{-1} (Sharova *et al* (2009); Friedel *et al* (2009)). A value outside this range was determined to enable the correct phase of *Cry1* and to be comparable to d_{Bmal1} as in Sharova *et al* (2009). d_{Cry1} is effecting only the *Cry1* phase and amplitude, since no regulation *via Cry1* is included in our model.
$d_{Ror\gamma}$	0.25	h^{-1}	Degradation rate of *Rorγ* mRNA	The experimentally determined value is 0.14 h^{-1} (Sharova *et al* (2009)). A value outside this range was determined to enable the correct phase of *Rorγ* and to be comparable to d_{Bmal1}, which was reported to have similar stability in Sharova *et al* (2009). $d_{Ror\gamma}$ effects only the *Rorγ* phase and amplitude, since no regulation *via Rorγ* is included in our model.

Symbol	Value	Unit	Description	Parameter notes
d_{Dbp}	0.45	h^{-1}	Degradation rate of *Dbp* mRNA	The experimentally determined range is 0.18 - 0.36 h^{-1} (Sharova *et al* (2009); Friedel *et al* (2009); Suter *et al* (2011)). A value outside this range was chosen to enable the correct phase. As with *Rev-erba*, RREs were discovered in Dbp regulatory region (Yamamoto *et al* (2004)), so some differential regulation is possible. Additionally, Sharova *et al* (2009) reported that the value of d_{Dbp} is between the values of d_{Bmal1} and $d_{Rev-erb\alpha}$. Increasing d_{Dbp} slightly increases period length and decreases all amplitudes except *Dbp*'s. It effects all phases; the effect is strongest on the phase of *Cry1*.
$bv2$	8		Factor for *Bmal1* activity on E/E'-boxes of *Rev-erba*	Increasing $bv2$ increases period length and normalised amplitudes of all genes. It affects mainly phase differences of *Cry1-Bmal1* and *Rev-erba-Bmal1*.
$bv3$	1		Factor for *Bmal1* activity on E/E'-boxes of *Per2*	Increasing $bv3$ up to the value of 1.7 decreases the period length and amplitudes of all genes. Beyond the value of 1.7, the period length decreases rapidly. It has the largest influence on the phase difference of *Rev-erba-Bmal1* and *Dbp-Bmal1*.
$bv4$	1		Factor for *Bmal1* activity on E/E'-boxes of *Cry1*	Increasing $bv4$ decreases the amplitude and influences the phase of *Cry1*.
$bv5$	1		Factor for *Bmal1* activity on E/E'-box of *Rorγ*	Increasing $bv5$ decreases the amplitude of *Rorγ* and slightly influences its phase.
$bv6$	10		Factor for *Bmal1* activity on E/E'-boxes of *Dbp*	Increasing $bv6$ beyond the default value slightly decreases the period and increases the amplitudes (except for *Rev-erba*). Its influence on phases is largest on *Per2*, *Cry1*, and *Rorγ*. In the range of values between 3 and 9, the period of the oscillations shortens to about 16 h and phases of the components change (especially of *Per2*, *Cry1*, and *Rorγ*).
$a1$	0.224			$\frac{a1}{ak1}$ represents the basal transcription rate of *Bmal1* through RRE. Increasing $a1$ and $ak1$ simultaneously without changing their ratio makes *Bmal1* expression less sensitive to regulation *via Rev-erba*. This decreases the period length and delays all genes compared to *Bmal1*. Increasing $a1$ increases the normalised amplitudes of all genes except *Dbp*.

Symbol	Value	Unit	Description	Parameter notes
$ak1$	0.26			Increasing $ak1$ decreases the period length and amplitudes of *Cry1*, *Bmal1*, and *Rorγ*. It affects mainly phase differences of *Cry1-Bmal1* and *Rorγ-Bmal1*. Beyond the value of 0.45, the period length shortens to about 16 h and phases of all genes change.
$a4$	0.327			$\frac{a4}{ak4}$ represents basal transcription rate of *Cry1* through RRE. Increasing $a4$ and $ak4$ simultaneously without changing their ratio makes *Cry1* expression less sensitive to regulation via *Rev-erba*, which causes *Cry1* to be expressed earlier and with smaller amplitude. Changing $a4$ in a range close to the default value has an effect on the absolute levels of *Cry1*, but not on the amplitude of its oscillation.
$ak4$	0.149			Increasing $ak4$ decreases the amplitude of *Cry1* oscillations and leads to its phase advance.
$a5$	0.405			$\frac{a5}{ak5}$ represents basal transcription rate of *Rorγ* through RRE. Increasing $a5$ and $ak5$ simultaneously without changing their ratio makes *Rorγ* expression less sensitive to regulation via *Rev-erba*, which causes *Rorγ* to be phase advanced and oscillating with lower amplitude. Changing $a5$ in a range close to the default value has an effect on the absolute levels of *Rorγ*, but not on the amplitude of its oscillation.
$ak5$	0.324			Increasing $ak5$ decreases the amplitude of *Rorγ* oscillations and leads to phase advance.
$c2$	1			$\frac{c2}{ck2} * \frac{b2}{bk2}$ represents basal transcription rate of *Rev-erba* through E/E'-boxes. Increasing $c2$ and $ck2$ simultaneously without changing their ratio makes *Rev-erba* less sensitive through regulation *via Per2*. That causes longer period length, larger amplitudes (except for *Cry1* and *Rorγ*), and earlier phases of all genes relative to *Bmal1*. Increasing $c2$ to the value of 1.3 increases the period length and amplitudes of all genes except *Per2*, *Cry1*, and *Rorγ*. It has the strongest effect on phases of *Dbp* and *Rev-erba* (relative to *Bmal1*). Beyond the value of 1.3, the period length jumps to about 16 h and phase distribution of all genes is different.
$ck2$	0.9			Increasing $ck2$ decreases the period length and amplitudes of *Dbp*, *Rev-erba*, and *Bmal1* the most. The largest influence on the phases is on the ones of *Per2*, *Dbp*, and *Rev-erba*. Decreasing $ck2$ below 0.3 changes the behaviour of the system (large decrease in period length, changes in amplitudes and phases of all genes).

Symbol	Value	Unit	Description	Parameter notes
$c3$	1.1			$\frac{c3}{ck3} * \frac{b3}{bk3}$ represents basal transcription rate of *Per2* through E/E'-boxes. Increasing $c3$ and $ck3$ simultaneously without changing their ratio makes *Per2* less sensitive to its auto-inhibition. This shortens the period, but the amplitudes are smaller and phases of all genes relative to *Bmal1* are delayed. When $c3$ and $ck3$ are larger than 1.4, period length shortens to 16 h and the overall behaviour of the system changes, although oscillations still persist. $c3$ has a strong influence on the whole system. Effect on the period length is variable, at values smaller than 1 the system changes considerably regarding the period and phases of all genes, but oscillations still persist.
$ck3$	1.1			Decreasing $ck3$ to the value of 0.44 decreases the period length, decreasing it even more increases the period length. It influences all amplitudes and the phase differences of *Cry1*, *Rorγ*, and *Per2* relative to *Bmal1*.
$c4$	1			$\frac{c4}{ck4} * \frac{b4}{bk4}$ represents basal transcription rate of *Cry1* through E/E'-boxes. Increasing $c4$ and $ck4$ simultaneously without changing their ratio increases *Cry1* amplitude and phase difference relative to *Bmal1*. Increasing c4 alone has no effect on *Cry1* phase and amplitude, but it influences absolute *Cry1* levels.
$ck4$	1.5			Increasing $ck4$ increases *Cry1* amplitude and phase difference relative to Bmal1.
$c5$	1.5			$\frac{c5}{ck5} * \frac{b5}{bk5}$ represents basal transcription rate of *Rorγ* through E/E'-boxes. Increasing $c5$ and $ck5$ simultaneously without changing their ratio decreases *Rorγ* amplitude and increases phase difference relative to *Bmal1*. Increasing $c5$ alone has no effect on *Rorγ* phase and amplitude, but it influences absolute *Rorγ* levels.
$ck5$	1			Increasing $ck5$ slightly decreases *Rorγ* amplitude and increases phase difference relative to *Bmal1*.
$c6$	0.75			$\frac{c6}{ck6} * \frac{b6}{bk6}$ represents basal transcription rate of *Dbp* through E/E'-boxes. Increasing $c6$ and $ck6$ simultaneously without changing their ratio makes *Dbp* less sensitive to inhibition via *Per2*, which has almost no effect on periods length and phases. If $c6$ and $ck6$ are both decreased below 0.6, the system changes considerably (period length of 16 h, different phases of most genes). Increasing $c6$ leads to longer period lengths. Decreasing it to below 0.6 causes large changes in the system regarding amplitudes, phase shifts between components, and period length.

Symbol	Value	Unit	Description	Parameter notes
$ck6$	0.75			Increasing $ck6$ to the value of 1.2 slightly increases period length and decreases the amplitudes of all genes. It mostly influences the phase of *Dbp*. Beyond the value of 1.2, the period length decreases rapidly and there are large changes in amplitudes and phases of all genes.
$b2$	1			$\frac{c2}{ck2} * \frac{b2}{bk2}$ represents basal transcription rate of *Rev-erba* through E/E'-boxes. Increasing $b2$ and $bk2$ in tandem without changing their ratio decreases period length and slightly decreases amplitudes of most genes. If they are both multiplied with factors smaller than 0.5, the system behaviour changes considerably (period length of about 18 h, smaller *Per2* amplitude, different phases of most genes).<br>Increasing $b2$ increases the period length and has an effect on all amplitudes (decrease in *Per2*, *Cry1*, and *Rorγ* amplitudes, increase in others). Increasing it beyond 1.3 has complex effects on the whole system (decrease of all amplitudes, changes of phases of all genes).
$bk2$	1.5			Increasing $bk2$ decreases period length and increases phase differences of all genes relative to *Bmal1*. At values smaller than 0.6, *Cry1* and *Rorγ* peaks are delayed.
$b3$	0.06			$\frac{c3}{ck3} * \frac{b3}{bk3}$ represents basal transcription rate of *Per2* through E/E'-boxes. Increasing $b3$ and $bk3$ in tandem without changing their ratio increases the period length and amplitudes of all genes. Multiplying both by a factor lower than 0.6 results in decreased amplitudes and changes in phases of *Per2*, *Cry1*, and *Rorγ*. Increasing $b3$ increases the period length and all amplitudes. Decreasing it below 0.04 has complex effects on the whole system, which results in lower amplitudes and different phases of all genes.
$bk3$	0.03			Increasing $bk3$ decreases the period length. For values larger than 0.05, there is a large difference in period length (it decreases to about 16 h) and phases of *Per2*, *Cry1*, and *Rorγ*.
$b4$	4			$\frac{c4}{ck4} * \frac{b4}{bk4}$ represents basal transcription rate of *Cry1* through E/E'-boxes. Increasing $b4$ and $bk4$ in tandem without changing their ratio decreases *Cry1* amplitude and increases its phase difference to *Bmal1*.<br>Increasing $b4$ alone increases *Cry1* amplitude and decreases its phase difference to *Bmal1*. It has no effect on period length.
$bk4$	1			Increasing bk4 decreases *Cry1* amplitude and delays it. It has no effect on period length.

Symbol	Value	Unit	Description	Parameter notes
$b5$	3.5			$\frac{c5}{ck5} * \frac{b5}{bk5}$ represents basal transcription rate of *Rorγ* through E/E'-boxes. Increasing $b5$ and $bk5$ in tandem without changing their ratio decreases *Rorγ* amplitude and increases its phase difference to *Bmal1*. Increasing $b5$ alone increases *Rorγ* amplitude. It has no effect on period length.
$bk5$	0.25			Increasing $bk5$ decreases *Rorγ* amplitude and delays it. It has no effect on period length.
$b6$	0.025			$\frac{c6}{ck6} * \frac{b6}{bk6}$ represents basal transcription rate of *Dbp* through E/E'-boxes. Increasing $b6$ and $bk6$ in tandem without changing their ratio slightly decreases the period length and amplitudes of all genes except *Per2*, where amplitude increases. The effect is largest on the change of phase of *Per2*. Multiplying both $b6$ and $bk6$ by a factor larger than 1.4 changes period length to about 16 h, decreases amplitude of *Per2*, and changes phase differences of all genes relative to *Bmal1*. Increasing $b6$ delays all genes and all amplitudes except *Dbp*, where amplitude decreases.
$bk6$	0.45			Decreasing $bk6$ decreases the period length and increases amplitudes of all genes. Increasing $bk6$ beyond 0.52 has complex effects on the system regarding period length, amplitudes, and phases of all genes.
$f2$	5			$\frac{f2}{fk2}$ represents the basal transcription rate of *Rev-erba* through D-boxes. Increasing $f2$ and $fv2$ in tandem without changing their ratio increases period length, has variable effect on amplitudes, and advances all genes relative to *Bmal1*. Increasing $f2$ beyond the value of 4 increases period length, has variable effects on amplitudes, and advances all genes relative to *Bmal1*. Decreasing $f2$ below 4 increases period length and amplitudes of *Dbp* and *Per2* (effect on other genes are opposite).
$fk2$	0.5			Increasing $fk2$ slightly increases period length, amplitudes of all genes except *Per2*, and phase difference relative to Bmal1 of all genes except *Per2* and *Dbp*.
$fv2$	1		Factor for *Dbp* activity on D-box of *Rev-erba*	Increasing $fv2$ increases the period and has variable small effects on amplitudes of most genes. It also influences phases of all genes; compared to *Bmal1*, *Per2*, *Dbp*, and *Rev-erba* are advanced and *Cry1* and *Rorγ* delayed.

Symbol	Value	Unit	Description	Parameter notes
$f3$	1			$\frac{f3}{fk3}$ represents the basal transcription rate of *Per2* through D-boxes. Increasing $f3$ and $fk3$ in tandem without changing their ratio decreases period length, amplitudes of all genes, decreases phase differences between *Cry1*-*Bmal1* and *Ror*γ-*Bmal1*, and increases the others. Increasing $f3$ decreases the period length and amplitudes of all genes. It has the largest effect on phase differences between *Cry1*-*Bmal1*, *Ror*γ-*Bmal1*, and *Per2*-*Bmal1*. Above the value of 1.4, the system changes considerably regarding period length, amplitudes, and phases.
$fk3$	0.1			Increasing $fk3$ slightly increases the period length and amplitudes of all genes. It has the strongest effect on phase difference between *Cry1* and *Bmal1*, but the effect is relatively small compared to some other parameters.
$fv3$	5		Factor for *Dbp* activity on D-box of *Per2*	$fv3$ modulates the effect of *Dbp* on D-box-mediated transcription of *Per2*. Increasing $fv3$ slightly increases the period length and amplitude of oscillations of all genes. It has a strong effect on phase difference between *Per2* and *Bmal1*.
$f4$	1			$\frac{f4}{fk4}$ represents the basal transcription rate of *Cry1* through D-boxes. Increasing $f4$ and $fk4$ in tandem without changing their ratio decreases *Cry1* amplitude and slightly phase difference between *Cry1* and *Bmal1*. Increasing $f4$ decreases *Cry1* amplitude and increases phase difference between *Cry1* and *Bmal1*.
$fk4$	1			Increasing $fk4$ decreases *Cry1* amplitude and phase difference between *Cry1* and *Bmal1*.
$fv4$	2		Modulation factor for *Dbp* activity on D-box of *Cry1*	Increasing $fv4$ slightly increases the *Cry1* amplitude and decreases phase difference between *Cry1* and *Bmal1*. Decreasing it below 1 increases *Cry1* amplitude and causes delayed phase with respect to *Bmal1*.

Table S7: Information on literature data of parameter values and comments on parameter estimation.

References

Friedel CC, Doelken L, Ruzsics Z, Koszinowski UH, Zimmer R (2009) Conserved principles of mammalian transcriptional regulation revealed by RNA half-life. *Nucleic Acids Res* **37**: e115

Hamilton EE, Kay SA (2008) SnapShot: circadian clock proteins. *Cell* **135**: 368–368.e1

Lee C, Etchegaray JP, Cagampang FR, Loudon AS, Reppert SM (2001) Posttranslational mechanisms regulate the mammalian circadian clock. *Cell* **107**: 855–867

Ogawa Y, Koike N, Kurosawa G, Soga T, Tomita M, Tei H (2011) Positive autoregulation delays the expression phase of mammalian clock gene Per2. *PLoS One* **6**: e18663

Preitner N, Damiola F, Lopez-Molina L, Zakany J, Duboule D, Albrecht U, Schibler U (2002) The orphan nuclear receptor REV-ERBalpha controls circadian transcription within the positive limb of the mammalian circadian oscillator. *Cell* **110**: 251–260

Rey G, Cesbron F, Rougemont J, Reinke H, Brunner M, Naef F (2011) Genome-wide and phase-specific DNA-binding rhythms of BMAL1 control circadian output functions in mouse liver. *PLoS Biol* **9**: e1000595

Ripperger JA, Schibler U (2006) Rhythmic CLOCK-BMAL1 binding to multiple E-box motifs drives circadian Dbp transcription and chromatin transitions. *Nat Genet* **38**: 369–374

Sharova LV, Sharov AA, Nedorezov T, Piao Y, Shaik N, Ko MSH (2009) Database for mRNA half-life of 19 977 genes obtained by DNA microarray analysis of pluripotent and differentiating mouse embryonic stem cells. *DNA Res* **16**: 45–58

Stratmann M, Stadler F, Tamanini F, van der Horst GTJ, Ripperger JA (2010) Flexible phase adjustment of circadian albumin D site-binding protein (DBP) gene expression by CRYPTOCHROME1. *Genes Dev* **24**: 1317–1328

Suter DM, Molina N, Gatfield D, Schneider K, Schibler U, Naef F (2011) Mammalian genes are transcribed with widely different bursting kinetics. *Science* **332**: 472–474

Ueda HR, Hayashi S, Chen W, Sano M, Machida M, Shigeyoshi Y, Iino M, Hashimoto S (2005) System-level identification of transcriptional circuits underlying mammalian circadian clocks. *Nat Genet* **37**: 187–192

Ukai-Tadenuma M, Yamada RG, Xu H, Ripperger JA, Liu AC, Ueda HR (2011) Delay in feedback repression by cryptochrome 1 is required for circadian clock function. *Cell* **144**: 268–281

Yamajuku D, Shibata Y, Kitazawa M, Katakura T, Urata H, Kojima T, Nakata O, Hashimoto S (2010) Identification of functional clock-controlled elements involved in differential timing of Per1 and Per2 transcription. *Nucleic Acids Res* **38**: 7964–7973

Yamamoto T, Nakahata Y, Soma H, Akashi M, Mamine T, Takumi T (2004) Transcriptional oscillation of canonical clock genes in mouse peripheral tissues. *BMC Mol Biol* **5**: 18
