# Supplementary material for: The Interplay of cis-Regulatory Elements Rules Circadian Rhythms in Mouse Liver
Source: PLoS One. 2012 Nov 5;7(11):e46835. doi: 10.1371/journal.pone.0046835 (PMC3489864; doi:10.1371/journal.pone.0046835)
Supplement: Supplementary Information S5 — Control analysis - robustness with respect to model parameters. Details on control analysis. (PDF) [file pone.0046835.s005.pdf]

S5 Control analysis - robustness with respect to model parameters

To analyse the behaviour of our model in response to small changes in parameter values, we performed a simplified control analysis, where we look for changes in system variables in response to relative changes in model parameters. Each model parameter was varied by ± 10 %. From simulations, properties such as period length, amplitudes, and phases of all genes relative to *Bmal1* peak were calculated. Supplementary Spreadsheet S1 shows relative changes in system variables as a percentage of the reference value (at default parameter value). For period length and phases, changes larger than 1 % are marked. For amplitudes, we put the limit at 3 %. Red and green refer to increasing and decreasing values, respectively.

Most influential parameters regarding period length are parameters belonging to *Per2*, the strongest being its explicit delay (τ_{Per2}, -7.3/7.6 % change of the period length for 10 % variation of the parameter), which is in agreement with analysis of one-variable and two-variable models. Also, parameters of *Bmal1* ($b3$, $bk3$, $bv3$) belonging to the *Per2* production term exhibit a strong effect on period length (about ± 1.5 % change in period length). The control of *Per2* phase is also crucial for phases and amplitudes. Besides parameters such as τ_{Per2} (2.5/-1.7 % change in phase of *Per2* relative to the phase of *Bmal1*) and d_{Per2} (2.3/-2.5 % change in phase of *Per2* relative to the phase of *Bmal1*), there are parameters belonging to *Per2* ($ck3$), *Bmal1* and *Rev-erba* (τ_{Bmal1}, $d_{Rev-erb\alpha}$) that influence *Per2* phase the most.

As *Per2* is important for the tuning of the system, the control of the *Per2* phase is also crucial for the behaviour of the system. The amplitudes are more sensitive than the period and phases for many of the parameters. The largest changes in the *Per2* amplitude can be achieved through the changing of the parameters that belong to *Per2* ($b3$, $bv3$, $ck3$, τ_{Per2} , d_{Per2}), and also through *Rev-erba* ($b2$, $d_{Rev-erb\alpha}$), *Bmal1* (d_{Bmal1}), and *Dbp* ($c6$, $bv6$).

Explicit delays show a strong influence on period length, phase control, and amplitudes of genes as discussed in the main text. For the sake of comparison to experimental results, additional parameters were included in our model. Parameter *transcription* multiplies all production terms with 0.9 or 1.1 and thus shows effects of transcription rate as explored in Dibner *et al* (2009). In our model, 10 % change in overall transcription rate results in 0.2 % change in period length. Although some phase changes are observed, the order of peaks remains unchanged. This is in agreement with findings from Dibner *et al* (2009) showing that circadian gene expression is resilient to fluctuations in overall transcription rates.

We additionally introduced parameters to vary the role of specific CCEs (E-box, D-box, or RRE). The corresponding values of tr_{E-box}, tr_{D-box}, and tr_{RRE} multiply each CCE's modulator term. These additional parameters have larger influence on the system as most of the individual parameters of our model. This infers that phase and amplitude determination of model components relies strongly on the interplay between different modulators (achieved through E-boxes, D-boxes, and RREs). Of all three CCEs, regulation through E-boxes has the largest effect on period length, phase, and amplitude determination.

Additionally, each gene's transcription term was multiplied by a certain factor (tr_{Bmal1}, $tr_{Rev-erb\alpha}$, tr_{Per2}, tr_{Cry1}, $tr_{Ror\gamma}$, tr_{Dbp}) to check for changes as a consequence of a change of solely one gene. In this case, changes in transcription of *Bmal1*, *Rev-erba* and *Per2* had the largest impact.

References

Dibner C, Sage D, Unser M, Bauer C, d'Eysmond T, Naef F, Schibler U (2009) Circadian gene expression is resilient to large fluctuations in overall transcription rates. *EMBO J* **28**: 123–134
