# Supplementary material for: The Interplay of cis-Regulatory Elements Rules Circadian Rhythms in Mouse Liver
Source: PLoS One. 2012 Nov 5;7(11):e46835. doi: 10.1371/journal.pone.0046835 (PMC3489864; doi:10.1371/journal.pone.0046835)
Supplement: Supplementary Information S6 — Primers. Additional information on RT-PCR and primers used in the study. (PDF) [file pone.0046835.s006.pdf]

S6 Primers

	**Gene name**	**NCBI acc. nr.**	**Primer**	**Primer sequence**	**Primer efficiency**
	Bmal1	NM_007489	fw	GCAGTGCCACTGACTACCAAGA	1.94
			rv	TCCTGGACATTGCATTGCAT	
	CKI-δ	NM_139059.2	fw	CCAGCCTGGAAGACCTGTTC	1.99
			rv	TGGCCAGCCCAAAGTCAA	
	CKI-ε	NM_013767.3	fw	GGATGTGAAGCCCGACAACTT	1.93
			rv	TCTCGACGGCTTTGCTCAAT	
	Clock	NM_007715.4	fw	CCTATCCTACCTTGGCCACACA	1.92
			rv	TCCCGTGGAGCAACCTAGAT	
	Cry1	NM_007771	fw	CCCAGGCTTTTCAAGGAATGGAACA	1.98
			rv	TCTCATCATGGTCATCAGACAGAGG	
	Cry2	NM_009963	fw	AGGGCTGCCAAGTGCATCAT	2.00
			rv	AGGAAGGGACAGATGCCAATAG	
	DBP	NM_016974.2	fw	AATGACCTTTGAACCTGATCCCGCT	1.94
			rv	GCTCCAGTACTTCTCATCCTTCTGT	
	Dec1	NM_011498.4	fw	TCTCCTACCCGAACATCTCAA	1.95
			rv	AATGCTTTCACGTGCTTCAA	
	Dec2	NM_024469.1	fw	ATTGCTTTACAGAATGGGGAGCG	1.89
			rv	AAAGCGCGCGAGGTATTGCAAGAC	
	E4bp4	NM_017373.3	fw	GGACCAGGGAGCAGAACC	1.95
			rv	GTCCGGCACAGGGTAAATC	
**Clock**	NONO	NM_023144.1	fw	CTGTGCCACCTGGTACTCC	1.87
**genes**			rv	CTTGGCCAAAACGTTCAGTT	
	Npas2	NM_008719	fw	CCCAGGAGTTACCAGTGCAG	1.91
			rv	GAGGAATGCAGAGCAGTCG	
	Per1	NM_011065.3	fw	TCCTCCTCCTACACTGCCTCT	1.90
			rv	TTGCTGACGACGGATCTTT	
	Per2	NM_011066.3	fw	CAACACAGACGACAGCATCA	1.94
			rv	TCCTGGTCCTCCTTCAACAC	
	Per3	NM_011067.1	fw	CTGCTCCAACTCAGCTTCCTTT	1.99
			rv	TTAGACAGCAAGGCTCTGGTTCT	
	Rev-erbα	NM_145434.1	fw	ACGACCCTGGACTCCAATAA	2.00
			rv	CCATTGGAGCTGTCACTGTAGA	
	Rev-erbβ	NM_011584.2	fw	ACGGATTCCCAGGAACATGG	1.93
			rv	CCTCCAGTGTTGCACAGGTA	
	RORα	NM_013646.1	fw	TTACGTGTGAAGGCTGCAAG	2.00
			rv	GGAGTAGGTGGCATTGCTCT	
	RORγ	NM_011281.2	fw	ACCTCTTTTCACGGGAGGA	1.93
			rv	TCCCACATCTCCCACATTG	
	SIRT1	NM_019812.1	fw	TCGTGGAGACATTTTTAATCAGG	1.83
			rv	GCTTCATGATGGCAAGTGG	

	Tim	NM_011589.1	fw	ACATGTGGGCAATGGCTT	1.78
			rv	CTGCTCCACAAAGTGAAAGGT	
	WDR5	NM_080848.2	fw	AAGAGCACAGCCCACTCCT	1.96
			rv	TTCAGGGCATAGTTTGGCTTA	
	Eif2A	NM_001005509.1	fw	CAACGTGGCAGCCTTACA	1.95
			rv	TTTCATGTCATAAAGTTGTAGGTTAGG	
**Reference genes**	Hmbs	NM_013551.2	fw	TCCCTGAAGGATGTGCCTA	1.64
			rv	AAGGGTTTTCCCGTTTGC	
	Ppib	NM_011149.2	fw	GGAGATGGCACAGGAGGAAA	1.93
			rv	CCGTAGTGCTTCAGTTTGAAGTTCT	

Table S8: Primer sequences and efficiencies. Additionally, primers for CIPC and Rorβ were constructed, but the transcripts were not detected in our mouse liver samples.
